# Impaired embryonic development in glucose-6-phosphate dehydrogenase-deficient *Caenorhabditis elegans* due to abnormal redox homeostasis induced activation of calcium-independent phospholipase and alteration of glycerophospholipid metabolism

**DOI:** 10.1038/cddis.2016.463

**Published:** 2017-01-12

**Authors:** Tzu-Ling Chen, Hung-Chi Yang, Cheng-Yu Hung, Meng-Hsin Ou, Yi-Yun Pan, Mei-Ling Cheng, Arnold Stern, Szecheng J Lo, Daniel Tsun-Yee Chiu

**Affiliations:** 1 Graduate Institute of Biomedical Sciences, College of Medicine, Chang Gung University, Taoyuan, Taiwan; 2 Department of Medical Biotechnology and Laboratory Sciences, College of Medicine, Chang Gung University, Taoyuan, Taiwan; 3 Healthy Aging Research Center, Chang Gung University, Taoyuan, Taiwan; 4 Metabolomics Core Laboratory, Chang Gung University, Taoyuan, Taiwan; 5 Clinical Phenome Center, Linkou Chang Gung Memorial Hospital, Taoyuan, Taiwan; 6 Graduate Institute of Medical Biotechnology and Laboratory Sciences, College of Medicine, Chang Gung University, Taoyuan, Taiwan; 7 Department of Biomedical Sciences, College of Medicine, Chang Gung University, Taoyuan, Taiwan; 8 New York University School of Medicine, New York,NY, USA; 9 Pediatric Hematology/Oncology, Linkou Chang Gung Memorial Hospital, Taoyuan, Taiwan

## Abstract

Glucose-6-phosphate dehydrogenase (G6PD) deficiency is a commonly pervasive inherited disease in many parts of the world. The complete lack of G6PD activity in a mouse model causes embryonic lethality. The G6PD-deficient *Caenorhabditis elegans* model also shows embryonic death as indicated by a severe hatching defect. Although increased oxidative stress has been implicated in both cases as the underlying cause, the exact mechanism has not been clearly delineated. In this study with *C. elegans*, membrane-associated defects, including enhanced permeability, defective polarity and cytokinesis, were found in G6PD-deficient embryos. The membrane-associated abnormalities were accompanied by impaired eggshell structure as evidenced by a transmission electron microscopic study. Such loss of membrane structural integrity was associated with abnormal lipid composition as lipidomic analysis revealed that lysoglycerophospholipids were significantly increased in G6PD-deficient embryos. Abnormal glycerophospholipid metabolism leading to defective embryonic development could be attributed to the increased activity of calcium-independent phospholipase A_2_ (iPLA) in G6PD-deficient embryos. This notion is further supported by the fact that the suppression of multiple iPLAs by genetic manipulation partially rescued the embryonic defects in G6PD-deficient embryos. In addition, G6PD deficiency induced disruption of redox balance as manifested by diminished NADPH and elevated lipid peroxidation in embryos. Taken together, disrupted lipid metabolism due to abnormal redox homeostasis is a major factor contributing to abnormal embryonic development in G6PD-deficient *C. elegans*.

The housekeeping gene glucose-6-phosphate dehydrogenase (G6PD), which is ubiquitously present in prokaryotic and eukaryotic organisms, encodes the rate-limiting enzyme in the pentose phosphate pathway. The importance of G6PD lies in the production of nicotinamide adenine dinucleotide phosphate (NADPH), which maintains intracellular redox homeostasis by regenerating glutathione in its reduced form.^1^ This is critical for negating the deleterious effects caused by excess oxidants including free radicals. NADPH is also required for the production of free radicals mediated by nitric oxide synthase and NADPH oxidase.^2^

Over 400 million individuals are inflicted by G6PD deficiency (favism) worldwide.^3^ A majority of G6PD mutations belong to single base missense mutation.^4^ The lack of reports on frameshift mutations or large deletions in this deficiency implies that complete loss of function of G6PD is incompatible with embryonic life.^5, 6^ Consistent with the report of embryonic lethality in G6PD-deficient mice, G6PD-deficient *Caenorhabditis elegans* displays impaired embryonic development indicated by severe hatching defects of embryos.^7^

Embryonic cell death induced by G6PD deficiency has been attributed to increased oxidative stress. G6PD-deficient embryonic stem cells fail to survive during oxidative stress.^8, 9, 10^ G6PD-knockout mice show embryonic arrest and death which is caused by oxidative damage.^5^ G6PD-knockdown zebrafish elicit embryonic defects and display enhanced oxidative stress-induced hemolysis upon treatment of pro-oxidants.^11^ These findings indicate an antioxidant role of G6PD during embryonic development. NADPH is also required for reductive biosynthesis, such as fatty acid synthesis and modification, during embryogenesis.^12, 13^ Lipids are essential in embryogenesis, and for example, perturbation in the regulation of membrane lipid metabolism causes early embryonic lethality in mouse.^14, 15^ How altered oxidative stress may affect lipid metabolism has largely been overlooked during embryonic development in G6PD-deficient organisms.

Lipidomics is a new omic technique aiming to globally analyze lipid species in a biological system. Such an approach can yield valuable information pertaining to the roles of lipids and establish metabolic pathways or networks that correlate with specific patho-physiological conditions.^16^ Phosphatidylcholine (PC) and phosphatidylethanolamine (PE) are the most abundant phospholipid species in eukaryotic cells, which account for more than half of the total phospholipids in eukaryotic membranes. Proper phospholipid composition is the key to establish and maintain the integrity of membrane structure and function. Phospholipids can be hydrolyzed by phospholipases, such as phospholipase A_2_ (PLA_2_).^17^ The PLA_2_ superfamily contains a diverse set of enzymes which cleave the sn-2 acyl bond of phospholipids and release a free fatty acid and a lysophospholipid. Both products of hydrolysis may produce second messengers that play essential roles in cellular signaling.^18, 19, 20^

We have made several novel discoveries. By using the cutting edge technology of lipidomics, we have demonstrated an abnormal lipid profile in G6PD-deficient *C. elegans* embryos with a marked increase in lysoglycerophospholipids. Such abnormal phospholipid composition is accompanied by the loss of membrane structural integrity as evidenced by electron microscopic studies and defective membrane properties, such as enhanced permeability, defective polarity and cytokinesis. Abnormal phospholipid metabolism associated with defective embryonic development could be attributed to the increased activity of calcium-independent phospholipase A_2_ (iPLA). This notion is corroborated by the partial rescue effect through the suppression of multiple iPLAs by genetic manipulation. Increased activity of iPLAs in G6PD-deficient embryos of *C. elegans* could be due to enhanced oxidative stress because diminished NADPH production and elevated lipid peroxidation have been detected in these embryos. This study provides the evidence for a potential mechanism of how G6PD deficiency causes embryonic lethality in *C. elegans*.

## Results

### Abnormal membrane function of embryos from G6PD-deficient *C. elegans*

#### Defective permeability of G6PD RNAi knockdown (Gi) embryos

**Figure 1:**
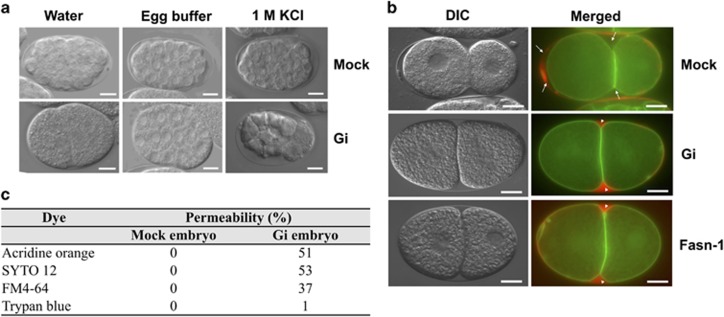
The impact of G6PD deficiency on *C. elegans* embryo physiology. (**a**) The effect of G6PD knockdown on *C. elegans* embryo morphology under physiological condition (Egg buffer) and osmotic stress (Water, KCl). Representative DIC images of embryos derived from Mock and G6PD(RNAi)(Gi) *C. elegans* are shown. White scale bar indicates 10 *μ*m. (**b**) The effect of G6PD knockdown and fatty acid synthase knockdown (Fasn-1(RNAi)) on permeability barrier formation in *C. elegans* embryos. Representative DIC and merged fluorescent images of two-cell embryos derived from Mock, Gi and Fasn-1(RNAi) of OD344 *C. elegans* are shown. In Mock embryo, the permeability barrier prevented the diffusion of red fluorescence (mCherry::CPG-2) to the surface of embryos (white arrow). The permeability barrier was disrupted in Gi embryos. Such inhibition caused the mCherry::CPG-2 to fill the space between the eggshell and embryo surface (white arrow head). Green fluorescence indicates the plasma membrane of the embryo. White scale bar indicates 10 *μ*m. (**c**) List of dye permeability ratio in Mock and Gi embryos with different dyes (*n*>100 embryos/group)

Previously, we have established the G6PD-deficient *C. elegans* model by feeding wild-type *C. elegans* with *Escherichia coli* expressing RNA-mediated interference (RNAi) targeting G6PD gene^7^ and nearly all embryos derived from G6PD-deficient *C. elegans* failed to hatch.^7^ The morphology of G6PD-deficient embryos was examined by microscopy (Figure 1). Unlike normal embryos, G6PD-deficient embryos displayed irregular, flattened and an egg-filling phenotype, indicating that the structure of G6PD-deficient embryos may be influenced by osmotic changes (Figure 1a). In high-salt solution, G6PD-deficient embryos displayed dramatic shrinkage indicated by crenated blastomeres. To determine the developmental stage, isolated embryos were stained with Hoechst 33342. Fluorescent microscopic images showed that over half of G6PD-deficient embryos (53%) were permeable to Hoechst 33342, while mock embryos were completely impermeable to the dye (Supplementary Figure S1a). To validate the observation, G6PD-deficient embryos were stained with trypan blue and other fluorescent dyes, including Acridine orange, SYTO12 and FM4-64 (Supplementary Figures S1b–e). G6PD-deficient embryos were permeable to all fluorescent dyes but not trypan blue (Figure 1c), indicating that the permeability defect of G6PD-deficient embryos was limited to small-molecule dyes.

#### Similar defective permeability of embryos from G6PD-deficient *C. elegans* and embryos from fatty acid synthase mutant *C. elegans*

Since the integrity of *C. elegans* eggshell and permeability barrier can be disrupted by metabolic alternations, such as inactivation of fatty acid synthesis, the permeability barrier in G6PD-deficient embryo was compared with fatty acid synthase-deficient embryo by using the permeability barrier reporter strain OD344 (mCherry::CPG-2;GFP::PH).^21^ The eggshell of a normal *C. elegans* embryo displayed a well defined structure, including an electron-dense thin outer vitelline layer, a middle chitin layer, a granule component layer, also known as chondroitin proteoglycan layer (CPG layer), and a perivitelline space.^21^ Inside the perivitelline space, the permeability barrier lies between the inner CPG layer and the plasma membrane. CPG-2 protein, extruded to the perivitelline space at the anaphase of meiosis I, is restricted outside the permeability barrier within the perivitelline space. The localization of mCherry::CPG-2 makes it a good fluorescent reporter for the integrity of the permeability barrier. The signal of mCherry::CPG-2 was detected at the circumference of the eggshell in normal embryo due to the intact permeability barrier, while mCherry::CPG-2 filled up the space between the eggshell and embryo plasma membrane in G6PD-deficient embryo (Figure 1b). This observation was similar to that observed in fatty acid synthase-deficient embryos. Fatty acid synthesis-derived permeability barrier formation has been demonstrated in the germline using the *rrf-1(pk1417)* mutant strain, where its germline RNAi is functional but the somatic RNAi is impaired.^21^ To determine whether the formation of permeability barrier modulated by G6PD occurred in somatic tissues or the germline, G6PD RNAi was performed in *ppw-1(pk1425)* mutant embryos that are sensitive to somatic RNAi, but resistant to germline RNAi. The results showed that half of G6PD-deficient embryos (51%) in the *ppw-1(pk1425)* background were permeable to Hoechst, while a portion of G6PD-deficient embryos (15%) in the *rrf-1(pk1417)* background were permeable to Hoechst, indicating that G6PD exerted broad effects on permeability mainly through somatic tissues with the germline playing a minor role.

#### A polarity defect of the two-cell stage of G6PD-deficient embryos

Phenotypes of G6PD-deficient embryos not only restricted to osmotic or permeability impairment, but also exhibited a polarity defect that was similar to mutants of fatty acid synthase having both polarity and osmotic defects (Figure 1b).^13, 22^ Embryos of wild-type N2 (*n*=33) and mock (*n*=32) showed normal first cell division, leading to a larger anterior cell (AB) and a smaller posterior cell (P_1_), indicating the asymmetric division. A subset of G6PD-deficient embryos (10%, *n*=60) had two daughter cells of equal size similar to *fasn-1* and *pod-2* mutants (33%, *n*=54), indicating symmetric division (polarity defect). As proper cell polarity plays a central role during early embryonic development, the early stage development of G6PD-deficient embryos was examined by time-lapse microscopy (Supplementary Figure S2). A large proportion of G6PD-deficient embryos (73%, *n*=45) showed the absence of cortical ruffling, pseudocleavage and an abnormal interaction of pro-nuclei with the cortex compared with mock embryos (summarized in Supplementary Table S1). Moreover, G6PD-deficient embryos took longer to develop or underwent embryonic arrest. These findings suggest that impairment in the beginning of embryogenesis was indicative of an early sign of embryonic lethality.

### Abnormal eggshell ultrastructure of G6PD-deficient embryos by transmission electronic microscopy

**Figure 2:**
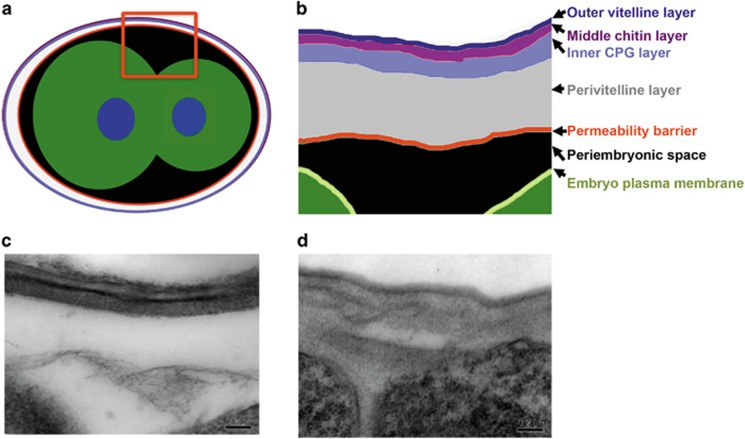
G6PD deficiency disrupts eggshell structure in *C. elegans* embryo indicated by TEM. (**a**) Schematic drawing of a normal *C. elegans* embryo. Red box indicates a portion of eggshell structure shown in (**b**). (**b**) Color-coded cartoon of the normal eggshell structure shown in (**c**). (**c**) Representative TEM image of the Mock embryo eggshell structure. (**d**) Representative TEM image of G6PD-deficient embryo eggshell structure. Black scale bar in (**c** and **d**) indicates 0.2 *μ*m

Since structure and function always go hand in hand, transmission electron microscopy (TEM) was used to examine the ultrastructure of the eggshell in G6PD-deficient embryo (Figure 2). The eggshell of G6PD-deficient embryo displayed a thin outer vitelline layer and a middle chitin layer similar to the normal eggshell. Two key differences were found in the eggshell of G6PD-deficient embryo: increased width of the electron-dense layer inside the chitin layer (probably the CPG layer). Unlike normal eggshell surrounded by a substantial perivitelline space, G6PD-deficient embryo showed a dramatic absence of such structure indicated by the close proximity of the CPG layer and the plasma membrane. The phenomenon of compressed or disappeared perivitelline space in G6PD-deficient embryo observed by TEM was consistent with the egg-filling phenotype observed by DIC microscopy (Figures 1a and b). G6PD-deficient embryo showed an abnormal but unique eggshell ultrastructure clearly distinguishable from normal embryo eggshell, strongly suggesting a role for G6PD in maintaining the normal structure of the eggshell permeability barrier.

### Altered lipid composition as shown by lipidomic analysis

#### Reliability of lipidomic analysis

**Figure 3:**
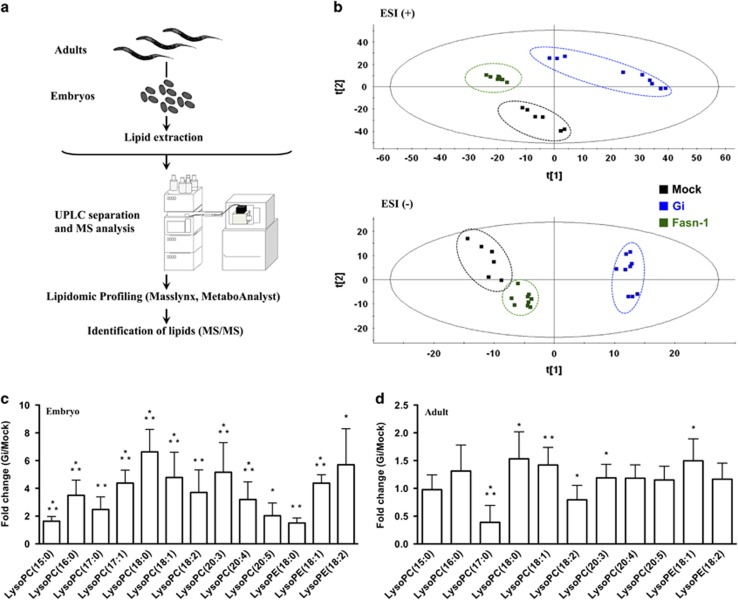
G6PD deficiency substantially increases lysoglycerophospholipids in *C. elegans* embryo as revealed by lipidomic analysis. (**a**) Workflow of lipidomic analysis. (**b**) The PCA plots of Mock, Gi and Fasn-1(RNAi) embryos in ESI^+^ and ESI^−^ mode. (**c**) Comparison of lysoglycerophospholipids between Mock and Gi embryos in ESI^+^ mode. (**d**) Comparison of lysoglycerophospholipids between Mock and Gi adults in ESI^+^ mode. All fold change data are presented as the mean±S.D. and statistical significance was calculated using a two-tailed *t*-test (*N*=3, *P<0.05; **P<0.005; ***P<0.001)

Since fatty acid biosynthesis is critical during embryonic development in *C. elegans*^13, 22, 23, 24^ and G6PD-deficient embryos displayed altered function of their membranes, the latter could be attributed to altered membrane lipids during embryogenesis. A lipidomic approach was carried out (Figure 3a and Supplementary Figure S3) to investigate whether G6PD deficiency affects lipid metabolism in *C. elegans*. Replicates of adults and embryos generated clusters in the principle component analysis (PCA) plot, suggesting that the analysis was reliable (Supplementary Figure S4). The lipidomic data were confirmed by feeding *C. elegans* with different *E. coli* strains (Supplementary Figures S5a and b). Mock embryos derived from parents fed with *E. coli* HT115 (Mock/HT115) and wild-type embryos derived from parents fed with *E. coli* OP50 (WT/OP50) formed separate clusters (Supplementary Figure S5a). Clusters of Mock/HT115 and wild-type embryos derived from parents fed with *E. coli* HT115 (WT/HT115) partially overlapped in the PCA plot (Supplementary Figure S5b). These data show that the lipidomic approach can distinguish altered lipidomic responses in embryos upon changing the bacterial diet. It also indicates that the lipidomic response of the Mock(RNAi) diet is analogous to that of the wild type with the same diet.

#### Altered lipidomic profile of G6PD-deficient embryos

Since G6PD-deficient *C. elegans* embryos phenocopied mutant embryos of fatty acid synthesis, their lipidomic profiles were compared (Figure 3b). In the PCA plot, G6PD-deficient embryos were well separated from Mock embryos in both the ESI^+^ and ESI^−^ modes. While *fasn-1*(RNAi) embryos were separated from Mock embryos in the ESI^+^ mode, it was closer to Mock embryos in the ESI^−^ mode. These distinct lipidomic profiles suggest that G6PD deficiency may modulate an alternative lipid metabolic pathway.

#### Increased lysoglycerophospholipids in G6PD-deficient embryos

Glycerophospholipids were identified as the main lipid class in *C. elegans* embryos (Table 1 and Supplementary Tables S2 and S3). Among glycerophospholipids, lysoPC and lysoPE were significantly enhanced in G6PD-deficient embryos (Figure 3c and Supplementary Table S4) but less in G6PD-deficient adults (Figure 3d). Long-chain lysoPCs, ranging from lysoPC(16 : 0) to lysoPC(20 : 5), were increased by 2-to 6-fold in G6PD-deficient embryos. Long-chain lysoPEs were increased by 1.5- to 5.7-fold in G6PD-deficient embryos. In contrast, most of the PCs were reduced in G6PD-deficient embryos (Table 1). Similarly, ether plasmalogens as well as branched chain fatty acid containing PEs were decreased in G6PD-deficient embryos (Table 1). In *fasn-1*(RNAi) embryos, the majority of long-chain lysoPCs and lysoPEs remained relatively constant (Supplementary Tables S2 and S3). The fact that G6PD deficiency not only induced defective membrane structure and function in the eggshell but also changed glycerophospholipid compositions in the embryo suggests that these G6PD-deficiency-induced abnormalities are closely linked during embryonic development.

### Inverse correlation between PLA_2_ activity and G6PD activity in *C. elegans* embryos

#### Increased PLA_2_ activity in G6PD-deficient embryos

**Figure 4:**
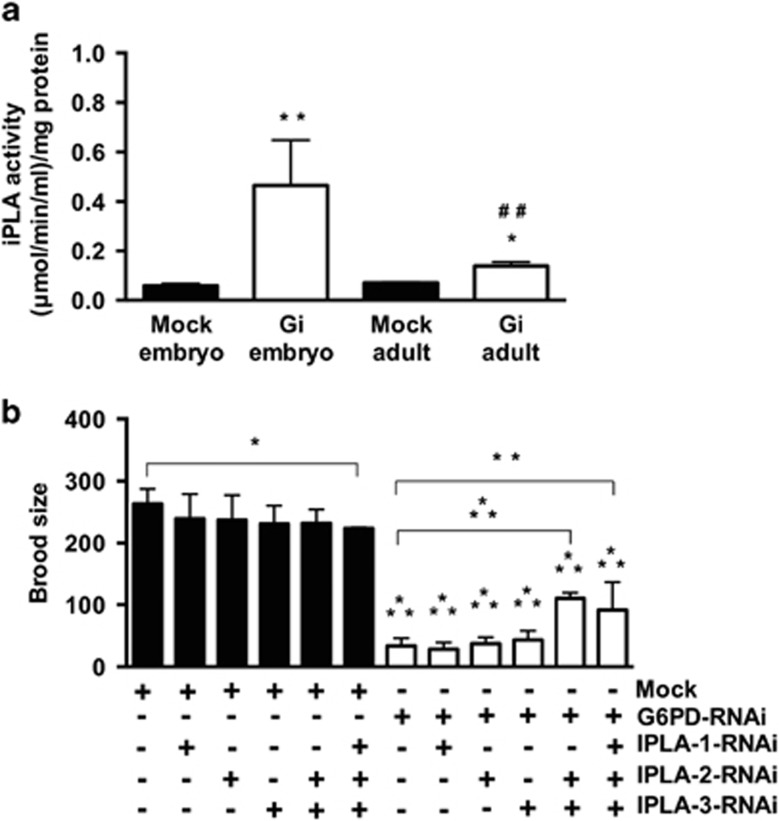
iPLA activity in G6PD deficiency-induced embryonic lethality. (**a**) The iPLA activity was determined in Mock and Gi adults (*N*=3, *P<0.05) and embryos (*N*=7, **P<0.005)(^##^*P*<0.005, Gi embryos compared with Gi adults). (**b**) Effect of iPLA inhibition on brood size (*N*>3, *n*>60 worms/experiment, *P<0.05; **P<0.005; ***P<0.001)

Based on the lipidomic analysis, it is postulated that during embryogenesis, G6PD regulates the activity of PLA_2_s, which are critical for organismal development.^25, 26, 27, 28, 29^ As iPLAs family was considered the main phospholipase in *C. elegans*,^25^ iPLA activity was determined (Figure 4a). Compared with mock, iPLA activity was increased in G6PD-deficient adults (2-fold, *P*<0.05) and even more so in G6PD-deficient embryos (10-fold, *P*<0.005). The iPLA activity of G6PD-deficient embryos was higher (3-fold, *P*<0.005) than that of G6PD-deficient adults. There was no significant difference (*P*>0.05) in the transcriptional level of iPLA in the adult, gonad or embryo between Mock and G6PD-deficient *C. elegans* (data not shown), indicating that G6PD deficiency stimulated iPLA protein activity. These results demonstrate that the iPLA activity was greatly enhanced in G6PD-deficient embryos and was consistent with the lipidomic data that lysoglycerophospholipids were dramatically altered in G6PD-deficient embryos (Figure 3c).

#### Alleviation of embryonic defects by suppression of the iPLA genes in G6PD-deficient embryos

Since G6PD deficiency enhanced the iPLA activity, it is reasonable to test whether the downregulation of iPLA could rescue defective phenotypes of G6PD-deficient embryos. Knockdown of the single iPLA gene (*ipla1, ipla2*, *ipla3*) failed to rescue the defective phenotypes of G6PD-deficient embryos (Figure 4b and Table 2). *C. elegans* shows a high degree of redundancy among *ipla* gene members in the genome, suggesting that each *ipla* member is likely involved in the remodeling of phospholipids.^25^ To avoid the potential redundant effects of *ipla* genes, the combination of RNAi targeting multiple *ipla* genes were used. The knockdown of two *ipla* genes (*ipla2* and *ipla3*) and three *ipla* genes (*ipla1*, *ipla2* and *ipla3*) significantly increased the brood size of G6PD-deficient *C. elegans* (Figure 4b). Although the downregulation of *ipla2* and *ipla3* was insufficient to rescue the permeability defect caused by G6PD deficiency, knockdown of three *ipla* genes alleviated such permeability defect (Table 2). No polarity defect was seen upon knockdown of the two *ipla* genes or all three *ipla* genes in G6PD-deficient embryos (Table 2). The beneficial effects of inhibition of multiple *ipla* genes by RNAi in G6PD deficiency supports the notion that G6PD deficiency enhanced iPLA activity which in turn altered the glycero phospholipid metabolism and membrane integrity during embryogenesis.

### Diminished NADPH production and association of increased iPLA activity with enhanced lipid peroxidation in G6PD-deficient embryos

**Figure 5:**
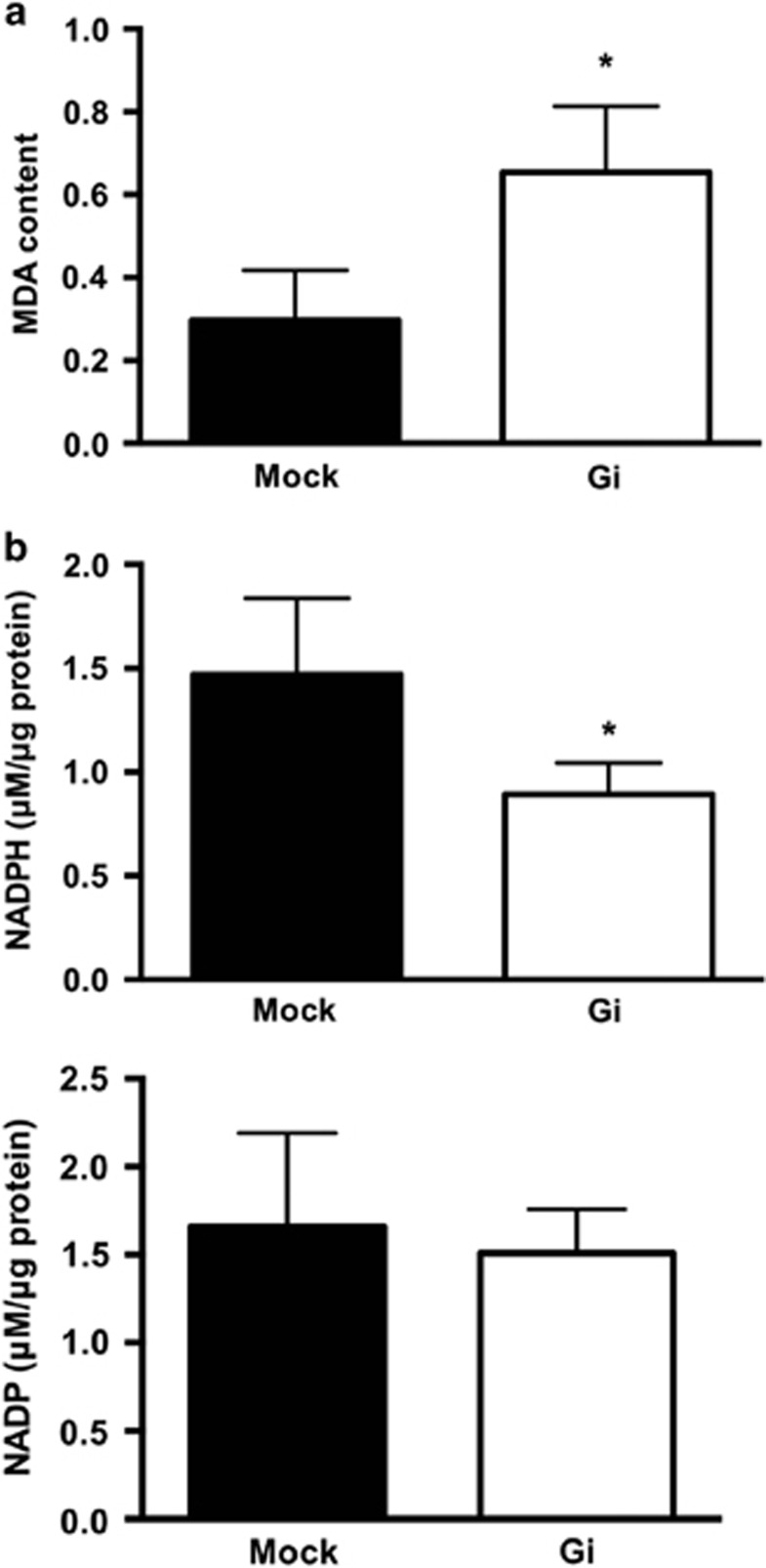
G6PD deficiency disrupts redox homeostasis by enhancing lipid peroxidation and reducing NADPH production. (**a**) Lipid peroxidation was determined in Mock and Gi embryos (*N*=4, *P<0.05). (**b**) NADPH and NADP levels were determined in Mock and Gi adults (*N*>3, *P<0.05)

Since PLA_2_ activity is associated with phospholipid peroxidation in cell membranes,^30^ the malondialdehyde (MDA) level was measured in embryos (Figure 5a). The MDA level was enhanced by 2-fold in G6PD-deficient embryos compared with mock embryos (Figure 5a). Such a finding is consistent with the observation that G6PD knockdown enhances oxidative stress and DNA oxidative damage in *C. elegans.*^7^ The level of NADP was not significantly altered (*P*=0.68) between Mock and G6PD-deficient adults (Figure 5b). However, the level of NADPH was decreased by 40% in G6PD-deficient adults compared with Mock adults. These findings indicate that G6PD deficiency disrupts redox homeostasis, thereby enhancing lipid peroxidation and activating phospholipase activity in *C. elegans* embryos.

## Discussion

The novel findings in this study provide evidence to explain why G6PD-deficient *C. elegans* has impaired embryonic development indicated by severe hatching defects reported previously.^7^ Reduced NADPH production and enhanced lipid peroxidation are associated with increased iPLA activity in G6PD-deficient embryos. Inhibition of multiple iPLAs by genetic manipulation can alleviate the embryonic impairment caused by G6PD deficiency. Increased lysoglycerophospholipids, revealed by lipidomics, is found in G6PD-deficient embryos, leading to abnormal membrane defects and eventually embryonic lethality.

G6PD is essential for growth and development in cells and organisms. G6PD plays a critical role in rapidly proliferating cells such as that during tumorigenesis^31, 32^ and during embryonic development.^5, 7, 11^ G6PD-deficient yeasts are viable but defective in methionine production.^33, 34^ Expression of acetaldehyde dehydrogenase restores the methionine auxotrophy in G6PD-deficient yeast, presumably by providing NADPH.^35^ These findings corroborate the classical antioxidant role of G6PD by providing cellular reducing equivalents, namely NADPH.

NADPH plays two major roles in cellular physiology. On the one hand, NADPH is an essential reducing power to maintain intracellular redox homeostasis by regenerating glutathione in its reduced form, which is critical for negating deleterious effects caused by excess oxidants including free radicals. On the other hand, NADPH is also required for reductive biosynthesis, including lipid synthesis and modification enzymes, for example, fatty acid synthase and cytochrome p450 reductase (CYP) EMB-8.^13^ Fatty acid biosynthesis is crucial for decision-making processes during embryonic development in *C. elegans*.^13, 21^ G6PD-deficient embryos phenocopy the membrane defects of fatty acid synthase mutant (Figure 1b), suggesting that G6PD is linked to membrane lipid metabolism during embryonic development. This notion is confirmed by the lipidomic analysis showing that glycerophospholipids are the major class of lipids being altered in G6PD-deficient embryos.

Alteration of the lipid metabolism impairs animal physiology.^36, 37^ The composition of fatty acids in phospholipids affects membrane structure.^38^ *C. elegans* can *de novo* synthesize a variety of polyunsaturated fatty acids (PUFA) with 18–20 carbons or obtain them from bacterial diet.^24^ Lipidomic analysis revealed that a majority of PUFA-containing lysoglycerophospholipids were increased in G6PD-deficient embryos (Supplementary Tables S2 and S3). The presence of PUFA in lysoPCs is consistent with previous *C. elegans* studies.^39, 40^ *C. elegans* CYPs utilize PUFA to generate eicosanoids, which are required for early embryonic development. Depletion of *C. elegans* CYPs causes defective embryonic phenotypes, including osmotic sensitivity, dye permeability and abnormal formation of permeability barrier.^13, 21^ G6PD-deficiency-induced permeability defects are similar to the embryo lacking chitin synthase or CPGs.^21^ Assembly of the eggshell requires exocytosis of ECM-modifying proteins, also called cortical granules, which facilitate the separation of the vitelline layer from the embryo surface and creates the perivitelline space.^41^ Within the cargoes of cortical granules, the CPG-1/2 is required for the formation of the permeability barrier. This barrier, formed during the anaphase of meiosis II, is an impenetrable envelope between the eggshell and the plasma membrane.^42^ The assumption that G6PD deficiency disrupts the formation of the permeability barrier is corroborated by the altered localization of mCherry::CPG-2 (Figure 1b) and the disrupted the eggshell ultrastructure (Figure 2). These results provide additional support to the notion that G6PD is required for the establishment of the permeability barrier in early embryonic development.

The permeability barrier is important for completion of meiosis and the establishment of polarity. A portion of G6PD-deficient embryos displayed a polarity defect and altered cytokinesis (Figure 1b and Supplementary Table S1). G6PD-deficient embryos phenocopy the polarity defect of the *C. elegans* mutant lacking essential enzymes of the fatty acid biosynthesis pathways, including FASN-1 and POD-2, as well as the fatty acid-modifying pathway, such as CYP-31A2 or CYP31A3.^13^ This phenomenon indicates that G6PD is linked to the establishment of cell polarity mediated by fatty acid synthesis and modification during embryonic development. Proper fatty acid composition is of paramount importance because it not only maintains membrane tension or curvature but also fosters a distinct membrane domain (microdomain or lipid raft) necessary for facilitating the interaction between the complex of the pronucleus/centrosome and cortex. Since fatty acids and lipid compositions are meticulously regulated in a given cell or tissue during development,^43^ the altered fatty acid composition in glycerophospholipids of G6PD-deficient embryos strongly suggests that G6PD is closely associated with the glycerophospholipids metabolism during embryonic development.

PLA_2_ plays an important role in membrane glycerophospholipid remodeling. The strongly elevated iPLA activity in G6PD-deficient embryos is in line with the increased lysoglycerophospholipids indicated by the lipidomic analysis (Figure 4a and Table 1). The differential level of iPLA activation between G6PD-deficient adults and embryos suggests that G6PD modulates iPLA activity during embryogenesis. Such a notion is not unprecedented, as choline kinase, a mammalian enzyme which phosphorylates choline to phosphocholine which is needed for PC synthesis, is crucial for embryogenesis but not required in the adult physiology in mouse.^15^ Conditional knockout of group VIC iPLA2 (PNPLA6) causes neurodegeneration, while complete inactivation of this gene leads to embryonic lethality,^44, 45^ confirming that iPLA is involved in embryonic development. The suppression of multiple iPLAs in G6PD-deficient *C. elegans* partially rescued the embryonic defects. This genetic manipulation indicates a strong correlation between G6PD status and iPLA activity in embryonic development.

Cellular metabolism is highly responsive to oxidative stress.^46, 47, 48^ The activation of iPLA is associated with oxidative damages, such as oxidized phospholipids.^49, 50^ Lipid peroxidation-stimulated PLA_2_ cleaves peroxidized fatty acids as a first step to repair oxidized phospholipids in membranes.^49, 50^ Lipid peroxidation also activates phospholipase C (PLC) in the rat brain homogenate, while reactive oxygen species scavengers block PLC activity and downstream signaling.^51^ Lipid hydroperoxides and their derivatives change the physical properties of cell membranes by altering phospholipid structure, orientation and dynamics.^52, 53^ Perturbation of red cell membrane by *t*-butyl hydroperoxide alters membrane phospholipid composition and permeability function.^54^ Since G6PD deficiency induces oxidative stress and oxidative damage,^6, 55^ the imbalanced redox environment, represented by decreased NADPH and elevated MDA due to G6PD deficiency, is likely responsible for the increased activity of iPLA. Intriguingly, the reduced NADPH level was found in G6PD-deficient *C. elegans*, while increased lysoglycerophospholipids were detected in their embryos. We reason that the main consequence of reduced NADPH level under G6PD deficiency is the induction of excess oxidative stress, which is indicated by the elevated lipid peroxidation in the embryo. Such lipid oxidative damage directly activates iPLA, which leads to the degradation of a fraction of PC or PE. Glycerophospholipids, including PC and PE, are the most abundant phospholipid species in eukaryotic cells (accounts for more than 50%), while the lysoglycerophospholipids are very low (<2%) due to their deleterious effect on the cell membrane. Thus, the increase of lysoPC or lysoPE derived from PC or PE is dramatic in G6PD-deficient embryos.

**Figure 6:**
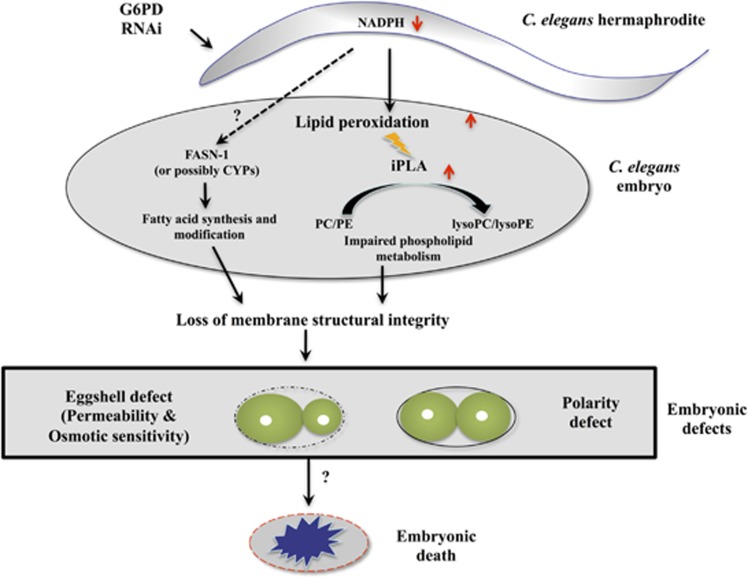
Proposed model of how G6PD deficiency causes embryonic lethality in *C. elegans*

Based on the evidence provided in this study, a scheme of G6PD deficiency-induced abnormal lipid metabolism leading to defective embryonic development in *C. elegans* is proposed (Figure 6). G6PD knockdown reduces NADPH production, which mainly impacts lipid metabolism through insufficient production of antioxidant capacity in embryos. As a result, elevated oxidized lipid products such as MDA and lipid peroxides in the zygote activate iPLA activity and generate excess production of lysoglycerophospholipids. Alternatively, decreased NADPH may modulate fatty acids synthesis through FASN-1 and possibly others such as CYPs. Together, disrupted lipid metabolism causes major structural and functional abnormalities of membranes leading to embryonic defects and eventually embryonic lethality.

## Materials and methods

### Nematode culture and RNAi silencing

N2 (wild type), *pod-2(ye60)*, *rrf-1(pk1417)*, *ppw-1(pk1425)* were acquired from Caenorhabditis Genetics Center (University of Minnesota, Minneapolis, MN, USA). The permeability barrier reporter strain OD344 was a gift from Prof. Sara Olson (Pomona College, Claremont, CA, USA). The nematode was propagated on a nematode growth medium (NGM) agar plate seeded with *E. coli* OP50 at 20 °C according to the standard protocols from Wormbook.^56^ For RNAi silencing experiments, synchronized L1 larvae by hypochlorite bleach were grown on NGM agar supplemented with 1 mM isopropyl-b-d-thiogalactopyranoside and antibiotics (ampicillin and carbenicillin) (Sigma-Aldrich, St Louis, MO, USA) and fed with HT115 *E. coli* harboring RNAi for silencing the specific gene target.^57^

### Phenotype assays

The morphology of embryos was examined by using embryos dissected from day 1 adults followed by mounting on a 2% agarose pad on a glass slide. DIC and fluorescent images were taken by using Leica DM 2500 (Leica, Ernst-Leitz-Straße, Wetzlar, Germany). For the osmotic sensitivity assay, embryos were placed in water, egg buffer or 1 M potassium chloride for 1 h at room temperature. For the dye permeability assay, embryos were stained with 0.4% trypan blue or 10 g/ml fluorescent dyes (Sigma-Aldrich) in PBS in the dark for 30 min. For the permeability barrier assay, embryos in the genetic background of OD344 (mCherry::CPG-2; GFP::PH) were used. The integrity of the permeability barrier was determined by the localization of mCherry::CPG-2. To quantify polarity, the area of two daughter cells was analyzed by Image J (NIH, Bethesda, MD, USA). The polarity defect was defined as the area of the anterior cell less than 53% of total embryonic cells. For brood size measurement, the total number of viable F1 worms derived from RNAi-treated parents was determined. For the embryonic development determination, time-lapsed images of embryos were taken at room temperature by using Leica DM 2500 (Leica).

### TEM

Gravid adults were frozen substituted in acetone (J.T. Baker, Phillipsburg, NJ, USA) containing 2% osmium tetroxide (Electron Microscopy Science, Hatfield, PA, USA) and 0.2% uranyl acetate (Electron Microscopy Science) in a Leica EM automatic freeze substitution machine (Leica). The temperature was raised from −90 °C to room temperature for 22 h (5 °C/h). The samples were dehydrated in 100% ethanol (Sigma-Aldrich) for 24 h, followed by two washes. The washed samples were embedded in the EMBed-812 embedding kit (Electron Microscopy Science) and incubated at 70 °C for 48 h. The 70 nm thin sections were cut by using a Reichert ultracut S microtome (Leica). Thin sections were collected on Formvar-coated copper grids (Electron Microscopy Science) and post-stained with 0.8% uranyl acetate (Electron Microscopy Science) in 40% methanol (J.T.Baker) followed by aqueous lead citrate (Electron Microscopy Science). Thin sections were viewed in a JEM-1230 transmission electron microscope (JEOL, Akishima, Tokyo, Japan).

### Lipidomic analysis

Replicates of *C. elegans* adult bearing eggs were washed off NGM plates by ultra water. The samples were lysed with bleach reagents (0.5 M NaOH and 1% NaOCl) for 5 min followed by wash with M9 buffer and centrifugation for three times. The mixture was filtered through a 12 × 75 mm BD Falcon cell-strainer cap (BD Biosciences, San Jose, CA, USA), centrifuged at 2500  r.p.m. for 5 min (Eppendorf, Hamburg, Germany) and washed with cold PBS for three times. Embryos were then subjected to lipid extraction using the Folch method.^58^ Lipidomic analysis was carried out using ultra-performance liquid-chromatography (UPLC) system (Waters, Milford, CT, USA) coupled with SYNAPT G1HDMS system (Waters). Chromatographic separation was performed on an Acquity CSH C18 column (particle size of 1.7 *μ*m, 2.1 mm × 100 mm; Waters). Column temperature was maintained at 55 °C. Chromasolv grade acetonitrile (ACN) and water solvents were obtained from Fluka (Sigma-Aldrich). For metabolite profiling, solvent A consisted of water :  ACN (60 : 40) with 10 mM ammonium formate (Sigma-Aldrich) and 0.1% formic acid (Sigma-Aldrich). Solvent B consisted of isopropanol (Sigma-Aldrich) : ACN (90 : 10) with 10 mM ammonium formate and 0.1% formic acid. The solvent gradient was as follows: 0–2 min, 40–43% solvent B; 2–2.1 min, 43–50% solvent B; 2.1–12 min, 50–54% solvent B; 12–12.1 min, 54–70% solvent B; 12.1–18 min, 70–99% solvent B; 18–20 min, 99–40% solvent B. A flow rate of 0.4 ml/min and an injected volume of 1.5 *μ*l of sample were used in ESI^+^, and 3 *μ*l of sample was used in ESI^−^. Mass spectrometric analysis was performed with the SYNAPT HDMS G1 system (Waters) in both ESI^+^ and ESI^−^ modes. The capillary and cone voltage were set at 3000 V (2000 V in ESI^−^ mode) and 30 V, respectively. The desolvation gas flow rate was set at 800 l/h. The desolvation and source temperatures were set at 400 and 100 °C, respectively. Mass spectrometric data were acquired over a range of *m*/*z* of 20–990 at rate of 0.1 per scan per second. Data were obtained in the centroid mode. The frequency of LockSpray was set at 0.5 s and corrected by averaging over 10 scans.

For data analysis, time-aligned ion features, mono-isotopic neutral mass, retention time as well and ion signal intensity were extracted from the raw data using the molecular feature extraction algorithm. MassLynx4.1 (Waters) and MetaboAnalyst were used for analysis and visualization of MS data sets in data metrices and PCA diagrams. Human Metabolome Database (http://www.hmdb.ca) and METLIN (http://metlin.scripps.edu/index.php) were used to search lipid metabolites. Candidates with high intensity were selected and validated by tandem mass spectrometry (MS/MS) with SYNAPT G1 HDMS system (Waters). Depending on the nature of compounds, the collision energy was ramped from 6 to 32 V. MS/MS spectra were acquired and confirmed by METLIN (http://metlin.scripps.edu/index.php) and LIPID MAPS (http://www.lipidmaps.org/). Metabolomic workflow of lipidomic profiling is listed in Supplementary Figure S3.

### Calcium-independent phospholipase A_2_ (iPLA) activity

The iPLA activity was determined by using the cPLA_2_ assay kit (Cayman Chemical, Ann Arbor, MI, USA). *C. elegans* embryos obtained by bleach were re-suspended in 50 mM HEPES (pH 7.4) containing 1 mM EDTA 300 *μ*l followed by sonication on ice (amplitude: 10%, 20 cycles of 2 s pulse and 5 s intervals) (Sonics and Materials, Newtown, CT, USA). The homogenates were centrifuged at 10 000 *g* for 15 min at 4 °C. The supernatants were collected for assay according to the manufacturer's protocol. The hydrolysis of the arachidonyl thio ester bond at the sn-2 position of synthetic substrate, arachidonyl thio-PC, releases a free thio, which can be detected by 5,5′-dithio-bis(2-nitrobenzoic acid) (DTNB). The activity of iPLA was determined in a calcium-free assay buffer (300 mM NaCl, 60% glycerol, 10 mM HEPES, 8 mM Triton X-100, 4 mM EGTA and 2 mg/ml of BSA). In brief, 10 *μ*l of samples or a positive control (Bee venom PLA_2_) were incubated with 5 *μ*l assay buffer. The reactions were initiated by adding 200 *μ*l substrate solution and incubated for 1 h at room temperature followed by the addition of 10 *μ*l of DTNB and incubation for 5 min at room temperature to stop reaction. The iPLA activity was calculated by the following formula and normalized for protein concentration:


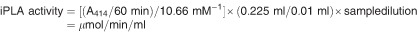


### MDA assay

MDA was determined by using the Oxiselect thiobarbituric acid reactive substances assay kit (Cell Biolabs Inc., San Diego, CA, USA). About 28 mg of *C. elegans* embryos was suspended in PBS containing 0.05% butylated hydroxyl toluene followed by sonication on ice. The homogenate was centrifuged at 10 000 *g* for 5 min. The supernatant (worm lysates) was collected for assay according to the manufacturer's protocol. In brief, 100 *μ*l of worm lysate or MDA standards were incubated with 100 *μ*l SDS lysis solution in separate microcentrifuge tubes at room temperature for 5 min. Each sample and standards were incubated with 250 *μ*l of 5.2 mg/ml TBA reagent (pH 3.5) at 95 °C for 60 min. The samples were placed in an ice bath for 5 min and centrifuged at 3000  r.p.m. for 15 min. The 200 *μ*l of supernatant of MDA standards or samples were transferred to a 96-well microplate and read by the absorbance spectroscopy at 532 nm. The MDA content in samples was determined by using the MDA standard curve. The results were normalized with the corresponding sample weight.

### NADPH measurement

The NADPH extraction method was modified from a previous protocol.^55^ *C. elegans* was collected and washed by M9 buffer on ice followed by transfer of the pellet into 2 ml Percellys tube (Bertin Technologies, Montigny-le-Bretonneux, France). Each tube was pre-filled with 100 *μ*l of 1 mm Ziconia beads (BioSpec Products, Bartlesville, OK, USA) and 1 ml of the extraction solvent containing 80% methanol (J.T.Baker) with 10 mM KOH which pre-cooled on ice. Samples were snap frozen in liquid nitrogen for 5 min and homogenized by Percellys 24 (Bertin Technologies) (6500  r.p.m., 30 s for two times, interval 5 s) at 4 °C. Samples were then held on ice for 15 min and centrifuged at 12 000  r.p.m., 4 °C for 15 min (Eppendorf). Supernatants were transferred to 15 mm × 45 mm screw thread clear vials (Thermo Fisher Scientific, Waltham, MA, USA) and pellets were re-extracted with 1 ml extraction solvent. Supernatant were air dried by nitrogen gas and stored at −80 °C. Re-suspended pellet with 300 *μ*l of water (Fluka) and centrifuged at 12 000  r.p.m. for 30 min for analysis. Samples were analyzed by UPLC equipped with a photodiode array detector (Waters). Samples were chromatographed on an Acquity HSST3 reversed-phase C18 column (particle size of 1.8 *μ*m, 2.1 mm × 150 mm; Waters). The composition of the mobile phase contained 25 mM potassium monobasic phosphate buffer (pH 6.0) (solvent A) and 100% methanol (solvent B) (J.T.Baker). The gradient was as follows: 0–2 min, 0% solvent B; 2–2.5 min, 0–3% solvent B; 2.5–5 min, 3–4% solvent B; 5–7 min, 4–15% solvent B; 7–8 min, 15% solvent B; 8–9 min, 15–0% solvent B; 9–11 min, 0% solvent B. The column temperature was maintained at 37 °C. The flow rate was set at 0.38 ml/min and an injected volume of 3 *μ*l of sample was used. Absorbance spectra were acquired from the wavelength at 260 and 340 nm. The NADPH contents were normalized by the protein concentration of the pellets. The protein was extracted by incubating pellets with 1 ml of 0.1 N NaOH at 65 °C for 30 min followed by determining protein concentration (Bradford).

### Statistical analysis

The LC-MS raw data were initially analyzed by Markerlynx (Waters). For modeling and reporting metabolomic data, the unsupervised PCA and orthogonal partial least-squares discriminant analysis (OPLS-DA) model and the Extended Statistics (EZinfo, Waters) were used. The variable importance in the projection (VIP) value of each variable in the model was calculated to represent its contribution to the grouping. A higher VIP value indicated a stronger contribution to discrimination among groups. Where applicable, presenting data were shown as mean±S.D. Statistical difference between mock and G6PD-deficient adults and embryos was analyzed by two-tailed *t*-test. Statistical tests were conducted using GraphPad Prism 6.0 (GraphPad Software, San Diego, CA, USA). Values of *P*<0.05 were considered statistically significant.

## Figures and Tables

**Table 1:** Altered lipid metabolites in G6PD-deficient *C. elegans* embryos

**Compound category**	**Identification**	***m/z***	**Formula**	**RT (min)**	**Mass error (Da)**	**Fold change (Gi/Mock)**	***P-*****value**	**VIP score**	**ESI**
Glycerophospholipids	LysoPC(15 : 0)	482.3256	C_23_H_48_NO_7_P	0.99	0.0015	1.63	<0.001	1.84	Positive
	LysoPC(16 : 0)^a^	496.3417	C_24_H_50_NO_7_P	1.17	0.0019	3.50	<0.001	2.37	Positive
	LysoPC(17 : 0)	510.3575	C_25_H_52_NO_7_P	1.31	0.0021	2.47	<0.005	2.10	Positive
	LysoPC(17 : 1)	508.3417	C_25_H_50_NO_7_P	1.10	0.0019	4.38	<0.001	2.28	Positive
	LysoPC(18 : 0)	524.3734	C_26_H_54_NO_7_P	1.60	0.0023	6.63	<0.001	2.43	Positive
	LysoPC(18 : 1)	522.3576	C_26_H_52_NO_7_P	1.21	0.0022	4.78	<0.001	2.28	Positive
	LysoPC(18 : 2)	520.3420	C_26_H_50_NO_7_P	1.00	0.0022	3.71	<0.005	2.27	Positive
	LysoPC(20 : 3)	546.3580	C_28_H_52_NO_7_P	1.08	0.0026	5.16	<0.001	2.31	Positive
	LysoPC(20 : 4)^a^	544.3418	C_28_H_50_NO_7_P	0.94	0.0020	3.19	<0.001	2.24	Positive
	LysoPC(20 : 5)^a^	542.3256	C_28_H_48_NO_7_P	0.84	0.0015	2.02	<0.05	1.49	Positive
	LysoPE(18 : 0)	482.3259	C_23_H_48_NO_7_P	1.69	0.0018	1.50	<0.005	1.68	Positive
	LysoPE(18 : 1)^a^	480.3108	C_23_H_46_NO_7_P	1.23	0.0023	4.37	<0.001	2.43	Positive
	LysoPE(18 : 2)^a^	478.3029	C_23_H_44_NO_7_P	1.05	0.0101	5.70	<0.05	2.37	Positive
	PC(19 : 1)	580.3636	C_27_H_54_NO_7_P	1.48	0.0016	5.53	<0.005	2.15	Negative
	PC(33 : 1)	746.5736	C_47_H_84_NO_8_P	6.35	0.0042	0.60	<0.001	2.06	Positive
	PC(33 : 1)	746.5728	C_41_H_80_NO_8_P	6.90	0.0034	1.20	<0.05	1.17	Positive
	PC(35 : 1)	774.6048	C_43_H_84_NO_8_P	9.17	0.0041	1.88	<0.005	1.99	Positive
	PC(35 : 1)	774.6046	C_43_H_84_NO_8_P	8.36	0.0039	0.60	<0.05	1.62	Positive
	PC(35 : 2)	772.5892	C_43_H_82_NO_8_P	6.76	0.0041	0.58	<0.001	1.81	Positive
	PC(35 : 2)	772.5892	C_43_H_82_NO_8_P	7.11	0.0041	0.85	<0.05	1.81	Positive
	PC(35 : 4)	768.5586	C_43_H_78_NO_8_P	4.93	0.0048	0.71	<0.05	1.79	Positive
	PC(35 : 5)	810.5307	C_43_H_76_NO_8_P	4.10	0.0016	0.74	<0.05	1.50	Negative
	PC(38 : 4)	810.6056	C_46_H_84_NO_8_P	7.22	0.0062	2.02	<0.001	2.32	Positive
	PC(38 : 5)	808.5906	C_46_H_82_NO_8_P	6.44	0.0055	1.74	<0.05	1.94	Positive
	PC(38 : 7)	848.5467	C_46_H_78_NO_8_P	4.08	0.0020	0.79	<0.05	1.20	Negative
	PC(38 : 8)	802.5426	C_46_H_76_NO_8_P	3.57	0.0045	0.63	<0.001	2.29	Positive
	PC(38 : 9)	800.5266	C_46_H_74_NO_8_P	3.08	0.0041	0.64	<0.001	2.04	Positive
	PC(39 : 5)	822.6067	C_47_H_84_NO_8_P	7.22	0.0060	0.51	<0.001	1.84	Positive
	PE(32 : 1)	690.5102	C_37_H_72_NO_8_P	6.19	0.0034	0.58	<0.001	2.37	Positive
	PE(33 : 1)	702.5096	C_38_H_74_NO_8_P	7.38	0.0017	0.86	<0.05	1.49	Negative
	PE(34 : 1)	718.5409	C_39_H_76_NO_8_P	8.19	0.0028	0.36	<0.001	2.20	Positive
	PE(34 : 2)	714.5089	C_39_H_74_NO_8_P	6.88	0.0010	0.62	<0.001	1.95	Negative
	PE(34 : 2)	716.5259	C_39_H_74_NO_8_P	6.94	0.0034	0.64	<0.001	2.12	Positive
	PE(35 : 1)	716.5620	C_40_H_78_NO_7_P	11.33	0.0031	0.24	<0.001	2.22	Positive
	PE(35 : 1)	718.5781	C_40_H_80_NO_7_P	12.72	0.0400	0.29	<0.001	2.12	Positive
	PE(36 : 1)	730.5782	C_41_H_80_NO_7_P	10.47	0.0037	0.23	<0.001	2.27	Positive
	PE(36 : 5)	736.4941	C_41_H_72_NO_8_P	5.18	0.0018	0.74	<0.05	1.42	Negative
	PE(37 : 1)	758.5732	C_42_H_82_NO_8_P	12.61	0.0027	1.13	<0.005	1.11	Negative
	PE(37 : 1)	746.6088	C_42_H_84_NO_7_P	13.02	0.0030	0.13	<0.001	2.33	Positive
	PE(37 : 2)	744.5934	C_42_H_82_NO_7_P	12.96	0.0032	0.11	<0.001	2.26	Positive
	PE(37 : 5)	752.5275	C_42_H_74_NO_8_P	5.77	0.0020	0.28	<0.001	1.85	Positive
	PE(37 : 5)	750.5092	C_42_H_74_NO_8_P	5.71	0.0013	0.45	<0.001	1.67	Negative
	PE(38 : 2)	756.5949	C_43_H_82_NO_7_P	12.61	0.0047	0.35	<0.001	2.07	Positive
	PE(P-36 : 1) or PE(O-36 : 2)	730.5777	C_41_H_80_NO_7_P	12.26	0.0032	0.34	<0.001	2.19	Positive
	PE(O-36 : 1) or PE(P-36 : 0)	732.5941	C_41_H_82_NO_7_P	12.61	0.0039	0.32	<0.001	2.03	Positive
	PE(P-36 : 2) or PE(O-36 : 3)	728.5625	C_41_H_78_NO_7_P	10.01	0.0036	0.51	<0.001	2.20	Positive
	PE(P-38 : 5) or PE(O-38 : 6)	748.5295	C_43_H_76_NO_7_P	7.78	0.0008	0.41	<0.001	1.88	Negative
									

Glycerolipids	TG(49 : 2)	834.7593	C_52_H_96_O_6_	15.31	0.0048	0.84	<0.05	1.70	Positive
	TG(50 : 3)	846.7602	C_53_H_96_O_6_	15.15	0.0057	0.77	<0.001	1.98	Positive
	TG(52 : 2)	876.8074	C_55_H_102_O_6_	15.69	0.0059	0.92	<0.05	2.01	Positive
	TG(53 : 2)	890.8234	C_56_H_104_O_6_	15.89	0.0063	1.27	<0.001	2.01	Positive
	TG(53 : 2)	895.7800	C_58_H_104_O_6_	15.89	0.0075	1.24	<0.005	1.85	Positive
	TG(55 : 6)	910.7956	C_58_H_100_O_6_	15.24	0.0098	2.12	<0.005	2.04	Positive

All metabolites in the list were verified by MS/MS in either ESI^+^ or ESI^−^ modes

a Confirmed by mass error. Others were confirmed by MS/MS and website database

**Table 2:** The effects of iPLA knockdown on permeability and polarity in *C. elegans* embryos

**Genotype**	**Permeability (%)**	**Polarity**
mock	0.0	0/30
*g6pd-1*(RNAi)	53.3±6.08	4/30
*ipla-1*(RNAi)	0.0	0/30
*ipla-1*(RNAi); *g6pd-1*(RNAi)	58.0±11.36	3/30
*ipla-2*(RNAi)	0.0	0/31
*ipla-2*(RNAi); *g6pd-1*(RNAi)	56.7±9.61	2/20
*ipla-3*(RNAi)	0.0	0/30
*ipla-3*(RNAi)*; g6pd-1*(RNAi)	49.7±1.53	1/20
*ipla-2*(RNAi); *ipla-3*(RNAi)	0.0	0/34
*ipla-2*(RNAi); *ipla-3*(RNAi); *g6pd-1*(RNAi)	50.3±5.51	0/22
*ipla-1*(RNAi)*; ipla-2*(RNAi); *ipla-3*(RNAi)	0.0	0/34
*ipla-1*(RNAi); *ipla-2*(RNAi); *ipla-3*(RNAi); *g6pd-1*(RNAi)	16.0±1.73^a^	0/36

a *P*<0.001compared with *g6pd-1*(RNAi)
