# Supplementary figures and images for: Impaired embryonic development in glucose-6-phosphate dehydrogenase-deficient Caenorhabditis elegans due to abnormal redox homeostasis induced activation of calcium-independent phospholipase and alteration of glycerophospholipid metabolism
Source: Cell Death Dis. 2017 Jan 12;8(1):e2545–. doi: 10.1038/cddis.2016.463 (PMC5386372; doi:10.1038/cddis.2016.463)

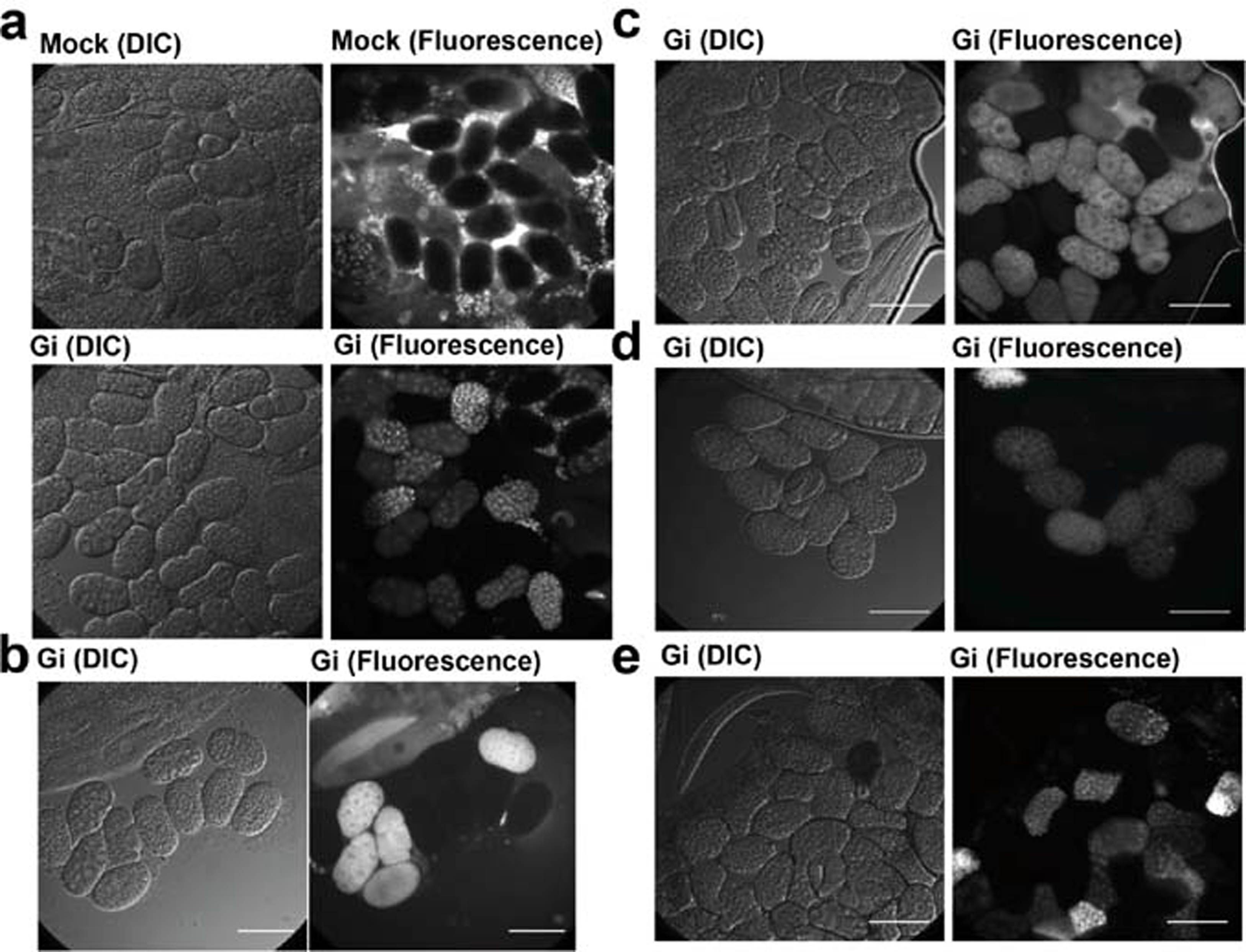

Supplement: Supplementary Figure S1 [file cddis2016463x1.tif]

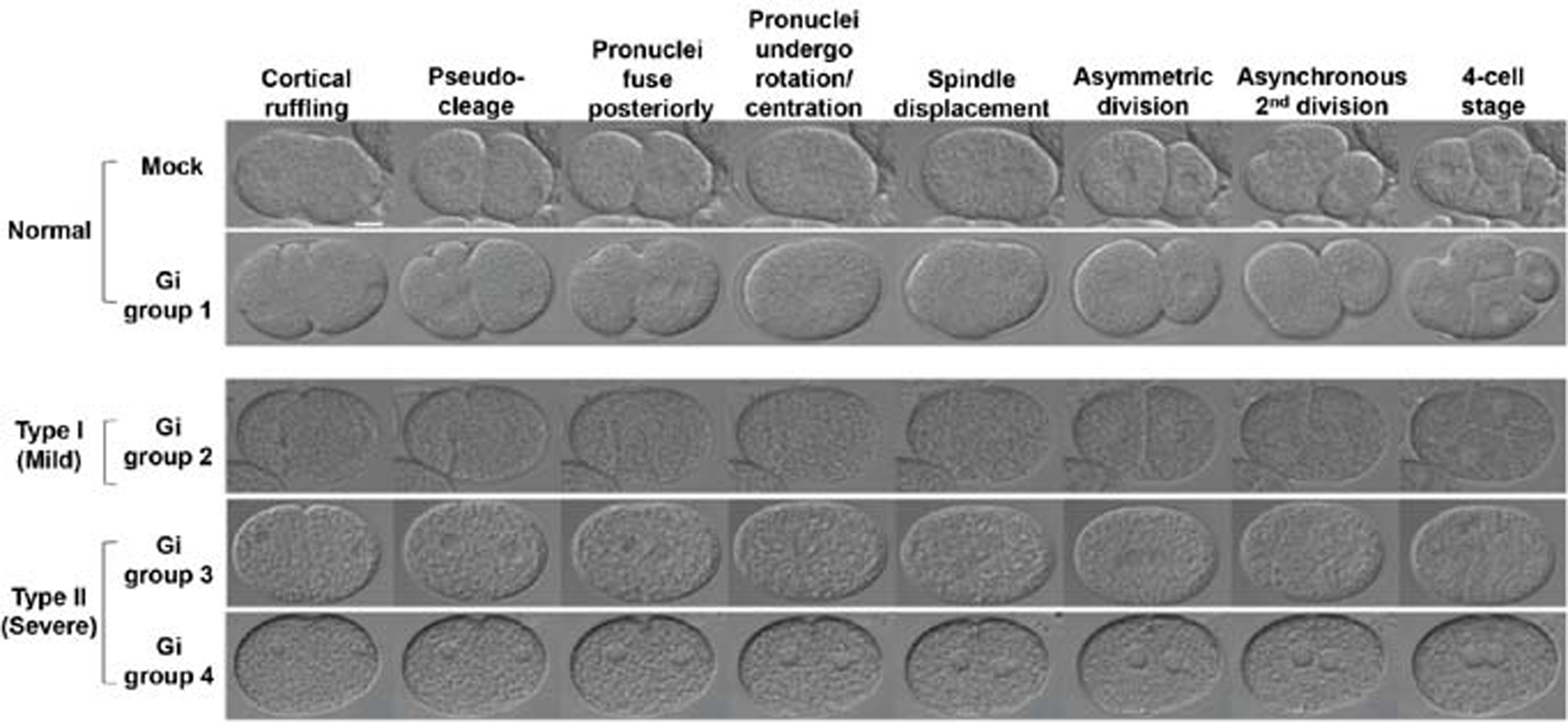

Supplement: Supplementary Figure S2 [file cddis2016463x2.tif]

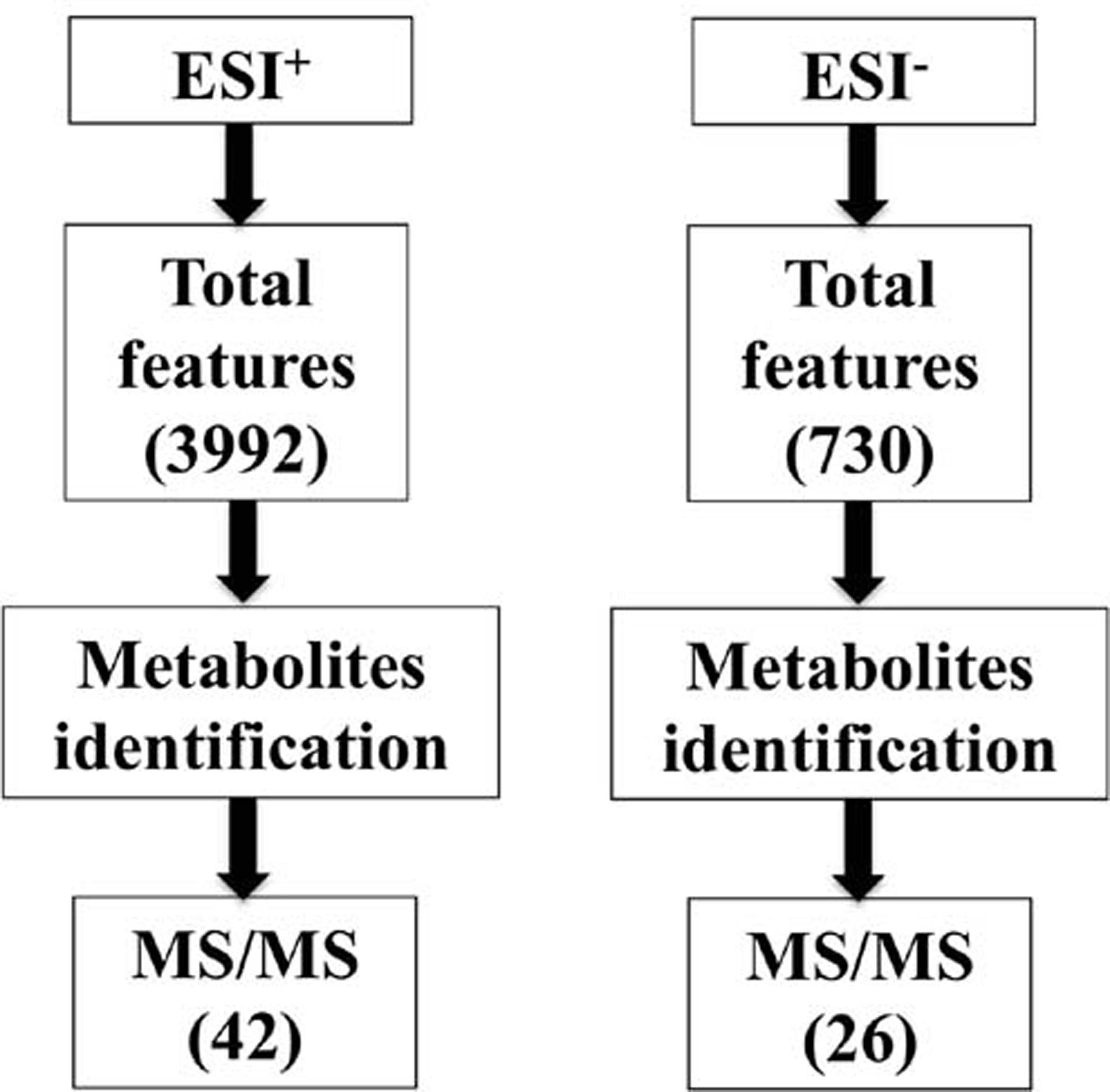

Supplement: Supplementary Figure S3 [file cddis2016463x3.tif]

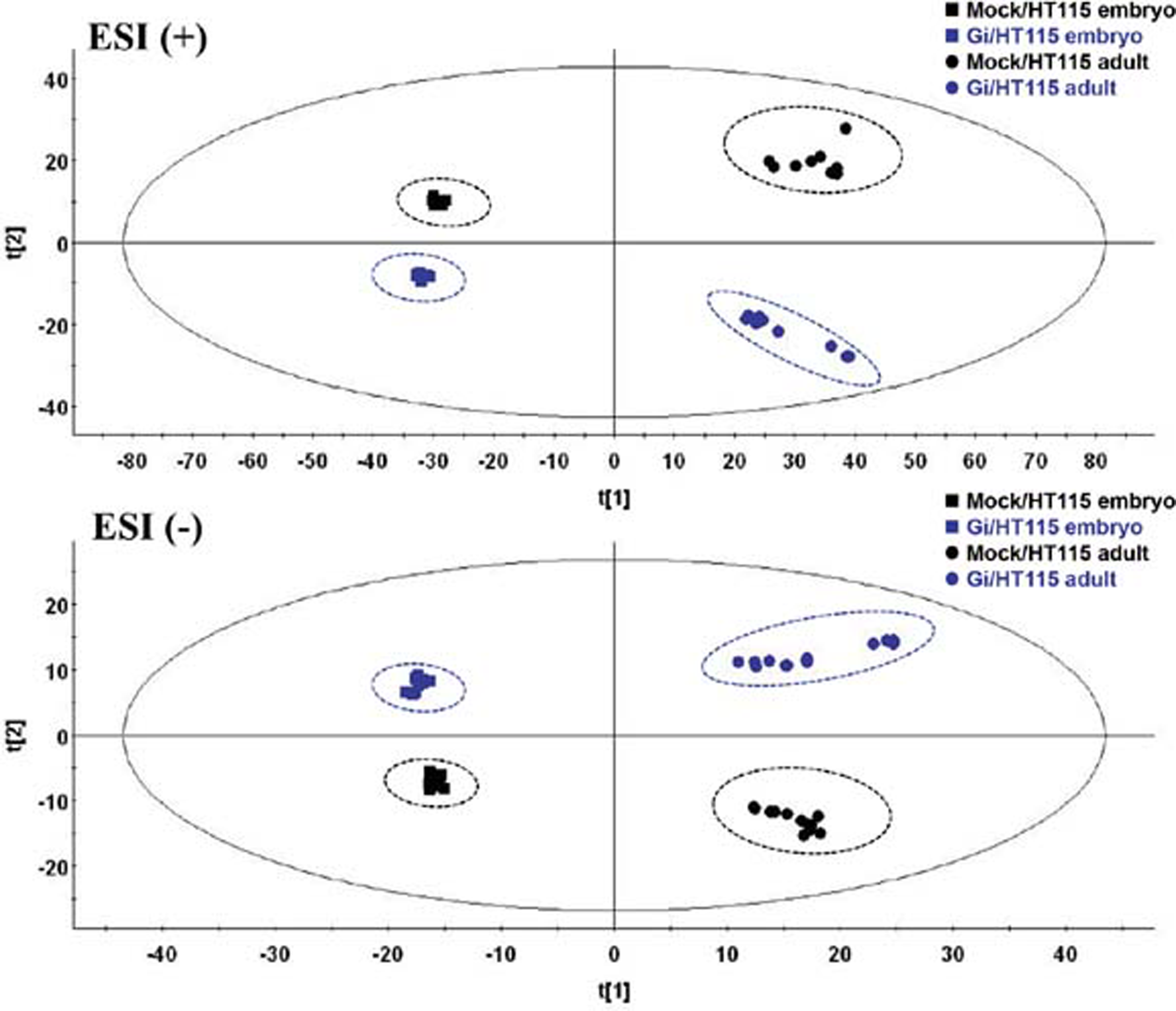

Supplement: Supplementary Figure S4 [file cddis2016463x4.tif]

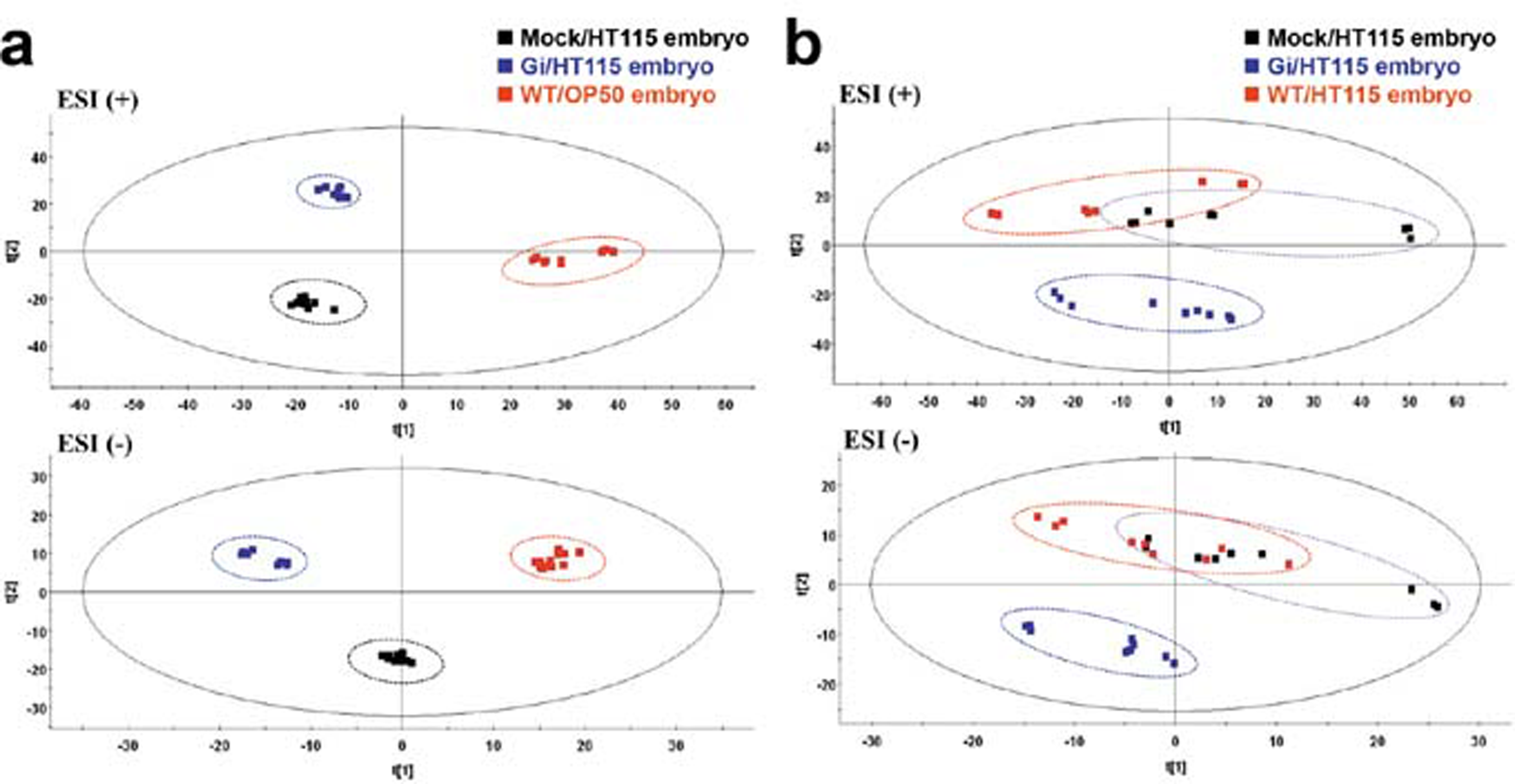

Supplement: Supplementary Figure S5 [file cddis2016463x5.tif]
