# Supplementary material for: Impaired embryonic development in glucose-6-phosphate dehydrogenase-deficient Caenorhabditis elegans due to abnormal redox homeostasis induced activation of calcium-independent phospholipase and alteration of glycerophospholipid metabolism
Source: Cell Death Dis. 2017 Jan 12;8(1):e2545–. doi: 10.1038/cddis.2016.463 (PMC5386372; doi:10.1038/cddis.2016.463)
Supplement: Supplementary Tables [file cddis2016463x6.doc]

| **Supplementary Table 1.** | | | | | |
| --- | --- | --- | --- | --- | --- |
| **Phenotypes** | **Mock** | **Gi group 1** | **Gi group 2** | **Gi group 3** | **Gi group 4** |
| Cortical ruffling | + | + | - | - | - |
| Pseudocleavage | + | + | - | - | - |
| Parental pronucleus associated closely with cortex | + | + | - | - | - |
| Pronuclei fused centrally | - | - | + | + | + |
| Pronuclei fused slowly | - | - | - | - | + |
| Asymmetric 1st division | + | + | - | NA | NA |
| Asynchronous 1st division | + | + | - | - | NA |
| Disorganized furrows | - | - | + | + | NA |
| Development time (1-cell to 2-cell stage) | 17 min | 20 min | 21 min | NA | > 40 min |
| Development time (1-cell to 4-cell stage) | 29 min | 33 min | 45 min | 40 min | NA |
| +, Present; -, Absence | | | | | |

| **Supplementary Table 2.** | | | | | | | | | | | |
| --- | --- | --- | --- | --- | --- | --- | --- | --- | --- | --- | --- |
| **Compound category** | **Identification** | **m/z** | **Formula** | **RT (min)** | **Mass error (Da)** | **Fold change (Gi/Mock embryo)** | ***P value* (Gi/Mock embryo)** | **Fold change (*fasn-1*(RNAi) /Mock embryo)** | ***P value* (*fasn-1*(RNAi)/ Mock embryo)** | **VIP score** | **ESI** |
| Glycerophos-pholipids |  |  |  |  |  |  |  |  |  |  |  |
|  | LysoPC(15:0) | 482.3256 | C_23_H_48_NO_7_P | 0.99 | 0.0015 | 1.63 | <0.001 | 1.40 | <0.05 | 1.84 | Positive |
|  | LysoPC(16:0)^⊹^ | 496.3417 | C_24_H_50_NO_7_P | 1.17 | 0.0019 | 3.50 | <0.001 | 1.62 | <0.05 | 2.37 | Positive |
|  | LysoPC(17:0) | 510.3575 | C_25_H_52_NO_7_P | 1.31 | 0.0021 | 2.47 | <0.005 | 1.65 | <0.005 | 2.10 | Positive |
|  | LysoPC(17:1) | 508.3417 | C_25_H_50_NO_7_P | 1.10 | 0.0019 | 4.38 | <0.001 | 1.67 | <0.005 | 2.28 | Positive |
|  | LysoPC(18:0)^⊹^ | 524.3734 | C_26_H_54_NO_7_P | 1.60 | 0.0023 | 6.63 | <0.001 | 2.43 | <0.001 | 2.43 | Positive |
|  | LysoPC(18:1) | 522.3576 | C_26_H_52_NO_7_P | 1.21 | 0.0022 | 4.78 | <0.001 | 2.82 | <0.001 | 2.28 | Positive |
|  | LysoPC(18:2) | 520.3420 | C_26_H_50_NO_7_P | 1.00 | 0.0022 | 3.71 | <0.005 | 1.95 | <0.05 | 2.27 | Positive |
|  | LysoPC(20:3) | 546.3580 | C_28_H_52_NO_7_P | 1.08 | 0.0026 | 5.16 | <0.001 | 2.12 | <0.005 | 2.31 | Positive |
|  | LysoPC(20:4)^⊹^ | 544.3418 | C_28_H_50_NO_7_P | 0.94 | 0.0020 | 3.19 | <0.001 | 1.71 | <0.05 | 2.24 | Positive |
|  | LysoPC(20:5)^⊹^ | 542.3256 | C_28_H_48_NO_7_P | 0.84 | 0.0015 | 2.02 | <0.05 | 1.40 | 0.133 | 1.49 | Positive |
|  | LysoPE(18:0) | 482.3259 | C_23_H_48_NO_7_P | 1.69 | 0.0018 | 1.50 | <0.005 | 0.80 | <0.05 | 1.68 | Positive |
|  | LysoPE(18:1)^⊹^ | 480.3108 | C_23_H_46_NO_7_P | 1.23 | 0.0023 | 4.37 | <0.001 | 2.27 | <0.001 | 2.43 | Positive |
|  | LysoPE(18:2)^⊹^ | 478.3029 | C_23_H_44_NO_7_P | 1.05 | 0.0101 | 5.70 | <0.05 | 2.11 | 0.228 | 2.37 | Positive |
|  | PC(33:1) | 746.5736 | C_47_H_84_NO_8_P | 6.35 | 0.0042 | 0.60 | <0.001 | 0.80 | <0.05 | 2.06 | Positive |
|  | PC(33:1) | 746.5728 | C_41_H_80_NO_8_P | 6.90 | 0.0034 | 1.20 | <0.05 | 0.58 | <0.001 | 1.17 | Positive |
|  | PC(35:1) | 774.6048 | C_43_H_84_NO_8_P | 9.17 | 0.0041 | 1.88 | <0.005 | 0.73 | 0.055 | 1.99 | Positive |
|  | PC(35:1) | 774.6046 | C_43_H_84_NO_8_P | 8.36 | 0.0039 | 0.60 | <0.05 | 0.84 | 0.416 | 1.62 | Positive |
|  | PC(35:2) | 772.5892 | C_43_H_82_NO_8_P | 6.76 | 0.0041 | 0.58 | <0.001 | 0.56 | <0.001 | 1.81 | Positive |
|  | PC(35:2) | 772.5892 | C_43_H_82_NO_8_P | 7.11 | 0.0041 | 0.85 | <0.05 | 0.69 | <0.001 | 1.81 | Positive |
|  | PC(35:4) | 768.5586 | C_43_H_78_NO_8_P | 4.93 | 0.0048 | 0.71 | <0.05 | 0.67 | <0.005 | 1.79 | Positive |
|  | PC(38:4) | 810.6056 | C_46_H_84_NO_8_P | 7.22 | 0.0062 | 2.02 | <0.001 | 0.90 | 0.343 | 2.32 | Positive |
|  | PC(38:5) | 808.5906 | C_46_H_82_NO_8_P | 6.44 | 0.0055 | 1.74 | <0.05 | 0.66 | <0.001 | 1.94 | Positive |
|  | PC(38:8) | 802.5426 | C_46_H_76_NO_8_P | 3.57 | 0.0045 | 0.63 | <0.001 | 0.60 | <0.001 | 2.29 | Positive |
|  | PC(38:9) | 800.5266 | C_46_H_74_NO_8_P | 3.08 | 0.0041 | 0.64 | <0.001 | 0.60 | <0.001 | 2.04 | Positive |
|  | PC(39:5) | 822.6067 | C_47_H_84_NO_8_P | 7.22 | 0.0060 | 0.51 | <0.001 | 0.28 | <0.001 | 1.84 | Positive |
|  | PC(39:5) | 822.6053 | C_47_H_84_NO_8_P | 6.73 | 0.0046 | 0.96 | 0.674 | 0.62 | <0.001 |  | Positive |
|  | PE(32:1) | 690.5102 | C_37_H_72_NO_8_P | 6.19 | 0.0034 | 0.58 | <0.001 | 0.71 | <0.001 | 2.37 | Positive |
|  | PE(34:1) | 718.5409 | C_39_H_76_NO_8_P | 8.19 | 0.0028 | 0.36 | <0.001 | 0.58 | <0.001 | 2.20 | Positive |
|  | PE(34:2) | 716.5259 | C_39_H_74_NO_8_P | 6.94 | 0.0034 | 0.64 | <0.001 | 0.49 | <0.001 | 2.12 | Positive |
|  | PE(34:2) | 716.5261 | C_39_H_74_NO_8_P | 6.61 | 0.0036 | 1.04 | 0.734 | 0.60 | <0.005 |  | Positive |
|  | PE(35:1) | 716.5620 | C_40_H_78_NO_7_P | 11.33 | 0.0031 | 0.24 | <0.001 | 0.63 | <0.001 | 2.22 | Positive |
|  | PE(35:1) | 718.5781 | C_40_H_80_NO_7_P | 12.72 | 0.0400 | 0.29 | <0.001 | 0.54 | <0.001 | 2.12 | Positive |
|  | PE(36:1) | 730.5782 | C_41_H_80_NO_7_P | 10.47 | 0.0037 | 0.23 | <0.001 | 0.66 | <0.001 | 2.27 | Positive |
|  | PE(37:1) | 746.6088 | C_42_H_84_NO_7_P | 13.02 | 0.0030 | 0.13 | <0.001 | 0.55 | <0.001 | 2.33 | Positive |
|  | PE(37:2) | 744.5934 | C_42_H_82_NO_7_P | 12.96 | 0.0032 | 0.11 | <0.001 | 0.38 | <0.001 | 2.26 | Positive |
|  | PE(37:5) | 752.5275 | C_42_H_74_NO_8_P | 5.77 | 0.0020 | 0.28 | <0.001 | 0.46 | <0.001 | 1.85 | Positive |
|  | PE(38:2) | 756.5949 | C_43_H_82_NO_7_P | 12.61 | 0.0047 | 0.35 | <0.001 | 0.57 | <0.001 | 2.07 | Positive |
|  | PE(P-36:2) or PE(O-36:3) | 728.5625 | C_41_H_78_NO_7_P | 10.01 | 0.0036 | 0.51 | <0.001 | 0.65 | <0.001 | 2.20 | Positive |
|  | PE(O-36:1) or PE(P-36:0) | 732.5941 | C_41_H_82_NO_7_P | 12.61 | 0.0039 | 0.32 | <0.001 | 0.75 | <0.05 | 2.03 | Positive |
|  | PE(P-36:1) or PE(O-36:2) | 730.5777 | C_41_H_80_NO_7_P | 12.26 | 0.0032 | 0.34 | <0.001 | 0.82 | 0.064 | 2.19 | Positive |
| Sphingolipids |  |  |  |  |  |  |  |  |  |  |  |
|  | Cer(d41:2) | 634.6171 | C_41_H_79_NO_3_ | 12.95 | 0.0038 | 0.66 | <0.05 | 0.75 | <0.05 |  | Positive |
| Glycerolipids |  |  |  |  |  |  |  |  |  |  |  |
|  | TG(49:1) | 836.7752 | C_52_H_98_O_6_ | 15.50 | 0.0050 | 1.52 | 0.453 | 1.33 | 0.608 |  | Positive |
|  | TG(49:2) | 834.7593 | C_52_H_96_O_6_ | 15.31 | 0.0048 | 0.84 | <0.05 | 0.90 | <0.05 | 1.70 | Positive |
|  | TG(50:3) | 846.7602 | C_53_H_96_O_6_ | 15.15 | 0.0057 | 0.77 | <0.001 | 0.92 | 0.076 | 1.98 | Positive |
|  | TG(52:2) | 876.8074 | C_55_H_102_O_6_ | 15.69 | 0.0059 | 0.92 | <0.05 | 1.18 | <0.005 | 2.01 | Positive |
|  | TG(53:2) | 890.8234 | C_56_H_104_O_6_ | 15.89 | 0.0063 | 1.27 | <0.001 | 1.15 | <0.05 | 2.01 | Positive |
|  | TG(53:2) | 895.7800 | C_58_H_104_O_6_ | 15.89 | 0.0075 | 1.24 | <0.005 | 1.15 | <0.001 | 1.85 | Positive |
|  | TG(55:6) | 910.7956 | C_58_H_100_O_6_ | 15.24 | 0.0098 | 2.12 | <0.005 | 0.65 | 0.199 | 2.04 | Positive |
| ^⊹^, confirmed by mass error. Others were confirmed by MS/MS and website database. | | | | | | | | | | | |

| **Supplementary Table 3.** | | | | | | | | | | | |
| --- | --- | --- | --- | --- | --- | --- | --- | --- | --- | --- | --- |
| **Compound category** | **Identification** | **m/z** | **Formula** | **RT (min)** | **Mass error (Da)** | **Fold change (Gi/Mock embryo)** | ***P value* (Gi/Mock embryo)** | **Fold change (*fasn-1*(RNAi)/ Mock embryo)** | ***P value* (*fasn-1*(RNAi) /Mock embryo)** | **VIP score** | **ESI** |
| Glycerophos-pholipids |  |  |  |  |  |  |  |  |  |  |  |
|  | LysoPC(16:0)^⊹^ | 540.3309 | C_24_H_50_NO_7_P | 1.17 | 0.0002 | 3.52 | <0.001 | 1.42 | 0.082 | 2.11 | Negative |
|  | LysoPC(17:1) | 552.3314 | C_25_H_50_NO_7_P | 1.10 | 0.0007 | 4.23 | <0.001 | 2.03 | <0.05 | 2.14 | Negative |
|  | LysoPC(18:0)^⊹^ | 568.3621 | C_26_H_54_NO_7_P | 1.60 | 0.0001 | 6.88 | <0.001 | 2.30 | <0.05 | 2.15 | Negative |
|  | LysoPC(18:1)^⊹^ | 566.3478 | C_26_H_52_NO_7_P | 1.21 | 0.0015 | 4.69 | <0.001 | 3.18 | <0.005 | 2.12 | Negative |
|  | LysoPC(18:2)^⊹^ | 564.3316 | C_26_H_50_NO_7_P | 1.00 | 0.0009 | 3.41 | <0.005 | 1.14 | 0.773 | 2.02 | Negative |
|  | LysoPC(20:3)^⊹^ | 590.3465 | C_28_H_52_NO_7_P | 1.09 | 0.0002 | 3.64 | <0.05 | 1.39 | 0.383 | 1.96 | Negative |
|  | LysoPC(20:4)^⊹^ | 588.3316 | C_28_H_50_NO_7_P | 0.95 | 0.0009 |  |  |  |  | 1.62 | Negative |
|  | LysoPC(20:5)^⊹^ | 586.3155 | C_28_H_48_NO_7_P | 0.83 | 0.0005 | 2.07 | <0.05 | 1.41 | 0.137 | 1.40 | Negative |
|  | LysoPE(18:0) | 480.3099 | C_23_H_48_NO_7_P | 1.69 | 0.0003 | 1.46 | <0.05 | 0.74 | <0.005 | 1.52 | Negative |
|  | LysoPE(18:1) | 478.2937 | C_23_H_46_NO_7_P | 1.22 | 0.0002 | 2.57 | <0.001 | 1.27 | <0.005 | 2.17 | Negative |
|  | LysoPE(18:2) | 476.2789 | C_23_H_44_NO_7_P | 1.00 | 0.0006 | 2.15 | <0.001 | 0.92 | 0.067 | 2.09 | Negative |
|  | PC(19:1) | 580.3636 | C_27_H_54_NO_7_P | 1.48 | 0.0016 | 5.53 | <0.005 | 2.52 | <0.05 | 2.15 | Negative |
|  | PC(35:5) | 810.5307 | C_43_H_76_NO_8_P | 4.10 | 0.0016 | 0.74 | <0.05 | 0.86 | 0.125 | 1.50 | Negative |
|  | PC(37:5) | 838.5620 | C_45_H_80_NO_8_P | 5.34 | 0.0016 | 0.60 | 0.087 | 0.68 | <0.05 |  | Negative |
|  | PC(37:6) | 836.5465 | C_45_H_78_NO_8_P | 4.55 | 0.0018 | 0.89 | 0.161 | 0.73 | <0.005 |  | Negative |
|  | PC(38:5) | 852.5784 | C_46_H_82_NO_8_P | 6.37 | 0.0024 | 1.48 | <0.001 | 0.83 | <0.05 | 1.87 | Negative |
|  | PC(38:7) | 848.5467 | C_46_H_78_NO_8_P | 4.08 | 0.0020 | 0.79 | <0.05 | 0.69 | <0.005 | 1.20 | Negative |
|  | PC(39:6) | 864.5785 | C_47_H_82_NO_8_P | 6.02 | 0.0025 | 0.23 | <0.005 | 0.56 | 0.322 |  | Negative |
|  | PE(32:1) | 688.4942 | C_37_H_72_NO_8_P | 6.13 | 0.0019 | 0.61 | <0.001 | 0.76 | <0.001 | 2.10 | Negative |
|  | PE(33:1) | 702.5096 | C_38_H_74_NO_8_P | 7.38 | 0.0017 | 0.86 | <0.05 | 0.63 | <0.001 | 1.49 | Negative |
|  | PE(34:1) | 716.5243 | C_39_H_76_NO_8_P | 8.13 | 0.0007 | 0.40 | <0.001 | 0.59 | <0.001 | 1.98 | Negative |
|  | PE(34:2) | 714.5089 | C_39_H_74_NO_8_P | 6.88 | 0.0010 | 0.62 | <0.001 | 0.57 | <0.001 | 1.95 | Negative |
|  | PE(36:5) | 736.4941 | C_41_H_72_NO_8_P | 5.18 | 0.0018 | 0.74 | <0.05 | 0.71 | <0.005 | 1.42 | Negative |
|  | PE(37:1) | 758.5732 | C_42_H_82_NO_8_P | 12.61 | 0.0027 | 1.13 | <0.005 | 0.64 | <0.001 | 1.11 | Negative |
|  | PE(37:1) | 742.5779 | C_42_H_82_NO_7_P | 12.81 | 0.0023 | 0.00 |  | 0.00 |  | 1.70 | Negative |
|  | PE(37:5) | 750.5092 | C_42_H_74_NO_8_P | 5.71 | 0.0013 | 0.45 | <0.001 | 0.63 | <0.005 | 1.67 | Negative |
|  | PE(38:5) | 764.5240 | C_43_H_76_NO_8_P | 6.84 | 0.0004 | 0.93 | 0.444 | 0.70 | <0.005 |  | Negative |
|  | PE(P-36:1) or PE(O-36:2) | 728.5609 | C_41_H_80_NO_7_P | 10.29 | 0.0009 | 0.15 | <0.05 | 0.13 | <0.05 |  | Negative |
|  | PE(P-36:1) or PE(O-36:2) | 728.5604 | C_41_H_80_NO_7_P | 12.11 | 0.0004 | 0.41 | <0.001 | 0.80 | 0.060 | 1.85 | Negative |
|  | PE(P-36:2) or PE(O-36:3) | 726.5448 | C_41_H_78_NO_7_P | 9.89 | 0.0005 | 0.27 | <0.001 | 0.55 | <0.001 | 1.95 | Negative |
|  | PE(P-38:0) or PE(O-38:1) | 730.5763 | C_41_H_82_NO_7_P | 12.59 | 0.0007 | 0.41 | <0.001 | 0.86 | <0.05 | 2.04 | Negative |
|  | PE(P-38:5) or PE(O-38:6) | 748.5295 | C_43_H_76_NO_7_P | 7.78 | 0.0008 | 0.41 | <0.001 | 0.60 | <0.001 | 1.88 | Negative |
| Sphingolipids |  |  |  |  |  |  |  |  |  |  |  |
|  | ceramide(d39:0) | 652.5886 | C_39_H_77_NO_3_ | 13.00 | 0.0001 | 1.17 | 0.062 | 0.76 | <0.005 | 1.00 | Negative |
| ^⊹^, confirmed by mass error. Others were confirmed by MS/MS and website database. | | | | | | | | | | | |

| **Supplementary Table 4.** | | | | | | | | | |
| --- | --- | --- | --- | --- | --- | --- | --- | --- | --- |
| **Compound category** | **Identification** | **m/z** | **Formula** | **RT (min)** | **Mass error (Da)** | **Fold change (Gi/Mock adult)** | ***P value* (Gi/Mock adult)** | **VIP score** | **ESI** |
| Glycerophos-pholipids |  |  |  |  |  |  |  |  |  |
|  | LysoPC(15:0)^⊹^ | 482.32 | C_23_H_48_NO_7_P | 0.98 | 0.0041 | 0.98 | 0.807 |  | Positive |
|  | LysoPC(16:0)^⊹^ | 496.34 | C_24_H_50_NO_7_P | 1.15 | 0.0001 | 1.31 | 0.082 | 1.00 | Positive |
|  | LysoPC(17:0)^⊹^ | 510.36 | C_25_H_52_NO_7_P | 1.28 | 0.0046 | 0.39 | <0.001 | 1.86 | Positive |
|  | LysoPC(18:0)^⊹^ | 524.37 | C_26_H_54_NO_7_P | 1.56 | 0.0011 | 1.53 | <0.05 | 1.53 | Positive |
|  | LysoPC(18:1)^⊹^ | 522.36 | C_26_H_52_NO_7_P | 1.19 | 0.0046 | 1.42 | <0.005 | 1.35 | Positive |
|  | LysoPC(18:2)^⊹^ | 520.34 | C_26_H_50_NO_7_P | 0.99 | 0.0002 | 0.79 | <0.05 | 1.18 | Positive |
|  | LysoPC(20:3)^⊹^ | 546.36 | C_28_H_52_NO_7_P | 1.05 | 0.0046 | 1.19 | <0.05 | 1.08 | Positive |
|  | LysoPC(20:4)^⊹^ | 544.34 | C_28_H_50_NO_7_P | 0.90 | 0.0002 | 1.18 | 0.051 | 1.12 | Positive |
|  | LysoPC(20:5)^⊹^ | 542.33 | C_28_H_48_NO_7_P | 0.83 | 0.0059 | 1.15 | 0.104 |  | Positive |
|  | LysoPE(18:1)^⊹^ | 480.31 | C_23_H_46_NO_7_P | 1.22 | 0.0015 | 1.50 | <0.05 | 1.63 | Positive |
|  | LysoPE(18:2)^⊹^ | 478.29 | C_23_H_44_NO_7_P | 0.99 | 0.0028 | 1.16 | 0.126 |  | Positive |
|  | PC(35:2)^⊹^ | 772.59 | C_43_H_82_NO_8_P | 6.34 | 0.0049 | 0.46 | <0.001 | 1.98 | Positive |
|  | PC(35:2)^⊹^ | 772.59 | C_43_H_82_NO_8_P | 6.60 | 0.0049 | 1.35 | <0.05 | 1.45 | Positive |
|  | PC(35:4)^⊹^ | 768.55 | C_43_H_78_NO_8_P | 4.63 | 0.0038 | 0.72 | <0.001 | 1.63 | Positive |
|  | PC(35:5)^⊹^ | 810.53 | C_43_H_76_NO_8_P | 4.04 | 0.0018 | 0.95 | 0.148 |  | Negative |
|  | PC(38:4)^⊹^ | 810.60 | C_46_H_84_NO_8_P | 6.78 | 0.0007 | 1.97 | <0.05 | 1.51 | Positive |
|  | PC(38:5)^⊹^ | 808.59 | C_46_H_82_NO_8_P | 6.05 | 0.0049 | 1.56 | <0.001 | 1.81 | Positive |
|  | PC(38:7)^⊹^ | 848.54 | C_46_H_78_NO_8_P | 4.00 | 0.0001 | 0.49 | <0.001 | 1.79 | Negative |
|  | PC(38:8)^⊹^ | 802.54 | C_46_H_76_NO_8_P | 3.41 | 0.0019 | 0.87 | <0.05 | 1.03 | Positive |
|  | PC(38:9)^⊹^ | 800.52 | C_46_H_74_NO_8_P | 2.98 | 0.0025 | 1.09 | 0.506 |  | Positive |
|  | PE(33:2)^⊹^ | 716.52 | C_39_H_74_NO_8_P | 6.19 | 0.0025 | 0.62 | <0.05 |  | Positive |
|  | PE(34:2)^⊹^ | 716.52 | C_39_H_74_NO_8_P | 6.53 | 0.0025 | 0.79 | 0.414 |  | Positive |
|  | PE(36:5)^⊹^ | 736.49 | C_41_H_72_NO_8_P | 5.08 | 0.0016 | 1.08 | <0.05 |  | Negative |
|  | PE(P-36:2) or PE(O-36:3)^⊹^ | 728.56 | C_41_H_78_NO_7_P | 9.31 | 0.0011 | 0.37 | <0.001 | 2.25 | Positive |
|  | PE(P-36:0) or PE(O-36:1)^⊹^ | 732.59 | C_41_H_82_NO_7_P | 11.89 | 0.0002 | 0.37 | <0.001 | 2.23 | Positive |
| Glycerolipids |  |  |  |  |  |  |  |  |  |
|  | TG(49:1)^⊹^ | 836.77 | C_52_H_98_O_6_ | 15.29 | 0.0002 | 0.48 | 0.108 |  | Positive |
|  | TG(49:2)^⊹^ | 834.76 | C_52_H_96_O_6_ | 15.07 | 0.0055 | 0.78 | 0.092 |  | Positive |
|  | TG(50:3)^⊹^ | 846.76 | C_53_H_96_O_6_ | 14.96 | 0.0055 | 1.34 | <0.001 | 1.86 | Positive |
|  | TG(52:2)^⊹^ | 876.81 | C_55_H_102_O_6_ | 15.48 | 0.0085 | 0.29 | <0.05 |  | Positive |
|  | TG(53:2)^⊹^ | 890.82 | C_56_H_104_O_6_ | 15.62 | 0.0029 | 0.18 | <0.05 | 1.09 | Positive |
|  | TG(55:6)^⊹^ | 910.79 | C_58_H_100_O_6_ | 15.01 | 0.0042 | 1.22 | <0.05 |  | Positive |
| ^⊹^, confirmed by mass error. Others were confirmed by MS/MS and website database. | | | | | | | | | |
