# Supplementary material for: Impaired embryonic development in glucose-6-phosphate dehydrogenase-deficient Caenorhabditis elegans due to abnormal redox homeostasis induced activation of calcium-independent phospholipase and alteration of glycerophospholipid metabolism
Source: Cell Death Dis. 2017 Jan 12;8(1):e2545–. doi: 10.1038/cddis.2016.463 (PMC5386372; doi:10.1038/cddis.2016.463)
Supplement: Supplementary Table and Figure Legends [file cddis2016463x7.docx]

**Supplementary Table legends**

Supplementary Table 1. Summary of early embryogenesis events affected by G6PD deficiency.

Supplementary Table 2. Lipid metabolites identified in Mock, Gi and *fasn-1*(RNAi) embryos in the ESI^+^ mode. All metabolites were verified by MS/MS. Depending on the nature of compounds, the collision energy was ramped from 6 V to 32 V.

Supplementary Table 3. Lipid metabolites identified in Mock, Gi and *fasn-1*(RNAi) embryos in the ESI^-^ mode. All metabolites were verified by MS/MS. Depending on the nature of compounds, the collision energy was ramped from 6 V to 32 V.

Supplementary Table 4. Lipid metabolites identified in Mock, Gi adults in the ESI^+^ and ESI^-^mode.

**Supplementary figure legends**

Supplementary Figure 1. The effect of G6PD knockdown on dye permeability in *C. elegans* embryo. **(a)** Representative DIC and fluorescent images of embryos derived from Mock and Gi *C. elegans* embryos are shown. **(b)** Representative DIC and fluorescent images of Gi embryos stained with Acridine orange. **(c)** Representative DIC and fluorescent images of Gi embryos stained with SYTO12. **(d)** Representative DIC and fluorescent images of Gi embryos stained with FM4-64. **(e)** Representative DIC and fluorescent images of Gi embryos stained with trypan blue and Hoechst 33342. White scale bar indicates 50 μm.

Supplementary Figure 2. Time-lapse images of Mock and Gi embryos. Representative images of mock and Gi group 1 were labeled with early embryogenesis hallmarks. Representative images of Gi group 2, 3 and 4 are shown in corresponding time course. Type I (mild) and type II (severe) of Gi embryos were categorized based on the degree of morphological alterations. White scale bar indicates 10 μm.

Supplementary Figure 3. The procedures of MS/MS analysis on *C. elegans* embryos. Total features of 3992 metabolites from the ESI^+^ mode and 730 metabolites from the ESI^-^ mode were analyzed for their variable importance in the projection (VIP) scores by Masslynx and Metaboanalyst. To identify the candidates, the resulting 848 metabolites from ESI^+^ mode and 198 metabolites from ESI^-^ mode with VIP scores > 1.0 were searched against the Human Metabolome Database (HMDB) and METLIN. To confirm the identification of the altered metabolites in Gi embryos, metabolites with relative abundance above 200 were verified by MS/MS. Depending on the nature of compounds, the collision energy was ramped from 6 V to 32 V.

Supplementary Figure 4. Lipidomic profiles of *C. elegans* embryos and adults fed with *E. coli* HT115. The principle component analysis (PCA) plot of Mock, Gi embryos and adults in the ESI^+^ mode and the ESI^-^ mode.

Supplementary Figure 5. Lipidomic profiles of *C. elegans* embryos derived from parents fed with different *E. coli* diet. The principle component analysis (PCA) plot of **(a)** WT/OP50, Mock, Gi embryos and **(b)** WT/HT115, Mock, and Gi embryos in the ESI^+^ mode and the ESI^-^ mode. WT/OP50: N2 fed with *E. coli* OP50, WT/HT115: N2 fed with *E. coli* HT115, Mock/HT115: N2 fed with *E. coli* HT115 bearing control RNAi, Gi/HT115: N2 fed with *E. coli* HT115 bearing G6PD RNAi.
